# Inhibition of mitochondrial respiration has fundamentally different effects on proliferation, cell survival and stress response in immature versus differentiated cardiomyocyte cell lines

**DOI:** 10.3389/fcell.2022.1011639

**Published:** 2022-09-23

**Authors:** Bent Grün, Michaela Tirre, Simon Pyschny, Vijay Singh, Hans-Gerd Kehl, Christian Jux, Jörg-Detlef Drenckhahn

**Affiliations:** ^1^ Department of Pediatric Cardiology, University Hospital Münster, Münster, Germany; ^2^ Department of Pediatric Hematology and Oncology, Justus Liebig University, Gießen, Germany; ^3^ Department of Pediatric Cardiology, Justus Liebig University, Gießen, Germany

**Keywords:** cardiomyocyte proliferation, cardiomyocyte survival, oxidative stress, mitochondrial dysfunction, cardiomyocyte differentiation, cellular stress response

## Abstract

Myocardial tissue homeostasis is critically important for heart development, growth and function throughout the life course. The loss of cardiomyocytes under pathological conditions ultimately leads to cardiovascular disease due to the limited regenerative capacity of the postnatal mammalian heart. Inhibition of electron transport along the mitochondrial respiratory chain causes cellular stress characterized by ATP depletion as well as excessive generation of reactive oxygen species. Adult cardiomyocytes are highly susceptible to mitochondrial dysfunction whereas embryonic cardiomyocytes in the mouse heart have been shown to be resistant towards mitochondrial complex III inhibition. To functionally characterize the molecular mechanisms mediating this stress tolerance, we used H9c2 cells as an *in vitro* model for immature cardiomyoblasts and treated them with various inhibitors of mitochondrial respiration. The complex I inhibitor rotenone rapidly induced cell cycle arrest and apoptosis whereas the complex III inhibitor antimycin A (AMA) had no effect on proliferation and only mildly increased cell death. HL-1 cells, a differentiated and contractile cardiomyocyte cell line from mouse atrium, were highly susceptible to AMA treatment evident by cell cycle arrest and death. AMA induced various stress response mechanisms in H9c2 cells, such as the mitochondrial unfolded protein response (UPR^mt^), integrated stress response (ISR), heat shock response (HSR) and antioxidative defense. Inhibition of the UPR, ISR and HSR by siRNA mediated knock down of key components does not impair growth of H9c2 cells upon AMA treatment. In contrast, knock down of NRF2, an important transcriptional regulator of genes involved in detoxification of reactive oxygen species, reduces growth of H9c2 cells upon AMA treatment. Various approaches to activate cell protective mechanisms and alleviate oxidative stress in HL-1 cells failed to rescue them from AMA induced growth arrest and death. In summary, these data show that the site of electron transport interruption along the mitochondrial respiratory chain determines cell fate in immature cardiomyoblasts. The study furthermore points to fundamental differences in stress tolerance and cell survival between immature and differentiated cardiomyocytes which may underlie the growth plasticity of embryonic cardiomyocytes during heart development but also highlight the obstacles of cardioprotective therapies in the adult heart.

## Introduction

Contractile cardiomyocytes rely on intact mitochondria due to their essential role in metabolic flux, energy generation, calcium handling and redox homeostasis. Consequently, mitochondrial dysfunction is involved in different cardiovascular pathologies, including mitochondrial cardiomyopathies, ischemia/reperfusion injury, drug induced cardiotoxicity, heart failure progression or myocardial ageing (Manolis et al., 2021). Apart from ATP depletion and imbalanced calcium flux, the most striking effect of mitochondrial dysfunction is the overproduction of reactive oxygen species (ROS) along the electron transport chain (ETC) (Zhou and Tian, 2018). The latter can lead to DNA, protein and lipid oxidation thereby impairing intracellular homeostasis and leading to oxidative stress. Whereas cells have a certain capacity for ROS detoxification to outlast periods of unfavorable growth conditions, prolonged or excessive oxidative damage can eventually result in cell death (Woo et al., 2021). Given the limited regenerative potential of the postnatal mammalian heart, loss of cardiomyocytes as a result of mitochondrial dysfunction contributes to impairment of heart function during various cardiovascular diseases.

It is widely recognized that mitochondrial dysfunction and oxidative stress can be detrimental for terminally differentiated cardiomyocytes in the adult heart (Peoples et al., 2019). The response of embryonic, fetal or neonatal cardiomyocytes towards mitochondrial dysfunction, however, is less clear. Given that embryonic and neonatal cardiomyocytes can proliferate and allow heart regeneration until the immediate postnatal period (Bongiovanni et al., 2021), it is tempting to speculate that they might also have a higher stress tolerance compared to adult cardiomyocytes. The combination of proliferative potential and a high level of cell protective signaling might equip the pre- and neonatal heart with the best possible plasticity to respond to various insults or pathologies during intrauterine and perinatal growth. Whether or not immature and terminally differentiated cardiomyocytes differ in their ability to deal with cellular stress seems to depend on the underlying conditions. Some studies have investigated the effect of doxorubicin, an anti-tumor agent known for its cardiotoxic side effects that are partially mediated by the impairment of mitochondrial function (Wallace et al., 2020). It has been shown that doxorubicin induces higher levels of programmed cell death (apoptosis) in neonatal compared to adult rat cardiomyocytes *in vitro* (Konorev et al., 2008) as well as in neonatal compared to juvenile mouse hearts *in vivo* (Shi et al., 2012). Indeed, advanced maturation seems to decrease the sensitivity towards doxorubicin induced cytotoxicity in human iPSC derived cardiomyocytes (Cui et al., 2019). On the other hand, human fetal cardiomyocytes have been proposed to exhibit lower apoptosis rates in response to simulated ischemia/reperfusion as compared to neonatal rat cardiomyocytes (Coles et al., 2005). Furthermore, immature rat cardiomyocytes form viable grafts when injected in injured myocardium whereas adult cardiomyocytes do not survive under the same conditions (Reinecke et al., 1999). Similarly, in rats engineered cardiac tissue from fetal cardiac cells engrafted onto infarcted adult left ventricles resulted in better LV contractility as compared to tissue derived from neonatal cells (Fujimoto et al., 2011). The latter was primarily attributed to proliferative activity, however, given that apoptosis was not different in fetal versus neonatal tissue grafts. Finally, dedifferentiation of adult cardiomyocytes in human ischemic myocardium or in the infarct border zone of sheep hearts is supposed to increase their stress resistance given that these cells show no signs of apoptosis (Ausma et al., 1998; Dispersyn et al., 2002). Such dedifferentiation results in the reactivation of fetal gene expression and even seems to allow cell cycle reentry (Kubin et al., 2011). In fact, reactivation of a fetal gene program in the adult heart under pathological conditions has been proposed to be protective by modulating cardiomyocyte metabolism and stress resistance (Rajabi et al., 2007).

We have previously shown that embryonic cardiomyocytes in the mid-gestational mouse heart survive mitochondrial dysfunction induced by loss of cytochrome c, an essential electron transporter between respiratory chain complex III and IV (Drenckhahn et al., 2008). Despite a reduced cell cycle activity of cytochrome c deficient cardiomyocytes during heart development, these cells are not eliminated from the myocardium via cell death and are even detectable in the neonatal and adult heart (Magarin et al., 2016). We identified a multitude of cell protective signaling pathways which are activated in embryonic cardiomyocytes upon loss of cytochrome c *in vivo*, including the mitochondrial unfolded protein response (UPR^mt^) (Shpilka and Haynes, 2018), the integrated stress response (ISR) (Pakos-Zebrucka et al., 2016) as well as various antioxidative and antiapoptotic mechanisms (Magarin et al., 2016). These findings confirmed an impressive plasticity of immature cardiomyocytes to cope with unfavorable oxidative stress conditions during intrauterine heart development. To further characterize the stress response of immature compared to differentiated cardiomyocytes we here applied an *in vitro* approach. We used H9c2 cells, an immature and non-contractile cardiomyoblast cell line from the embryonic rat heart (Kimes and Brandt, 1976; Hescheler et al., 1991), and HL-1 cells, a differentiated and contractile cardiomyocyte cell line from the murine atrium (Claycomb et al., 1998), and treated them with different pharmacological inhibitors of the mitochondrial respiratory chain. A special focus lied on the complex III inhibitor antimycin A, as it best resembles the loss of cytochrome c in our previous *in vivo* studies. We monitored differences in cell death and survival, cell cycle activity and proliferation as well as activation of stress response signaling between H9c2 and HL-1 cells subjected to mitochondrial dysfunction. In addition, we aimed to identify molecular mechanisms that are required for survival of immature cardiomyocytes and at the same time might be able to protect differentiated cardiomyocytes from mitochondrial stress.

## Materials and methods

### Cell culture

H9c2 cells were purchased from ATCC (CRL-1446) and cultured in DMEM (FG0445) containing 10% fetal bovine serum (FBS) (S0115) and 1% penicillin/streptomycin (all components from Biochrom) at 37°C with 95% air and 5% CO_2_. For characterization of optimal culture conditions upon ETC inhibitor treatment the following FBS batches were compared: F7524 Batch number BCBV7611 (FBS 1 in Supplementary Figure S2C) and BCCC6626 (FBS 2 in Supplementary Figure S2C), F2442 Batch number 15K234 (FBS 3 in Supplementary Figure S2C, all from Sigma Aldrich). For studying the effect of glucose availability DMEM containing high (D6429) and low (D6046, both from Sigma Aldrich) glucose concentration were compared.

HL-1 cells were purchased from Merck/Sigma Aldrich (SCC065) and cultured as originally described (Claycomb et al., 1998). Culture dishes or wells were coated with 5 μg/ml Fibronectin (F1141) in 0.02% gelatin (G9391) solution at 37°C over night. Cells were seeded in 12 well plates (8 × 10^4^ cells per well) or 6 well plates (2 × 10^5^ cells per well) and cultured in Claycomb Medium (51800C) containing 10% fetal bovine serum (F2442), 10 µM norepinephrine [A0937, dissolved as 10 mM stock in 30 mM ascorbic acid (A7506)], 2 mM L-glutamine (G7513) and 1% penicillin/streptomycin at 37°C with 95% air and 5% CO_2_. All components were purchased from Sigma Aldrich. Norepinephrine was omitted from the culture medium for subsequent treatment with ETC inhibitors or small molecules.

### Reagents and treatments

The following ETC inhibitors, reagents and small molecules were used for cell treatment throughout the study (final concentrations are provided in the respective figures or figure legends): Antimycin A dissolved in DMSO (A8674, Sigma Aldrich (different lots); sc-202467, Santa Cruz; ab141904, Abcam), Rotenone dissolved in DMSO (R8875, Sigma Aldrich), Oligomycin dissolved in DMSO (O4876, Sigma Aldrich), trans-ISRIB dissolved in DMSO (16258, Cayman Chemical), Salubrinal dissolved in DMSO (SML0951, Sigma Aldrich), Celastrol dissolved in DMSO (C0869, Sigma Aldrich), Geranylgeranylacetone dissolved in DMSO (G5048, Sigma Aldrich), CDDO Methyl Ester dissolved in DMSO (SMB00376, Sigma Aldrich), N-Acetyl-L-cysteine dissolved in H_2_O (A7250, Sigma Aldrich), Sodium pyruvate dissolved in H_2_O (P5280, Sigma Aldrich), L-Aspartic acid dissolved in 1 M HCl (A7219, Sigma Aldrich), H_2_O_2_ diluted in PBS (9681, Carl Roth), DMSO (A3672, AppliChem).

### Evaluation of cell growth and cytotoxicity

Cell growth and cytotoxicity were evaluated by MTT [3-(4,5-Dimethylthiazol-2-yl)-2,5-diphenyl-tetrazoliumbromid, M2128, Sigma Aldrich] assays. Cells were seeded in 12 well plates with 5 × 10^4^ cells per well. After 24 h the starting cell mass (t0) was evaluated before the addition of any reagents. 0.5 mg/ml MTT in cell culture medium was added to the wells and incubated for 1.5 h at 37°C. Medium was removed and cells were lysed with 40 mM HCl in isopropanol for 5 min. The cell lysate was homogenized, centrifuged to remove cell debris and absorbance of the supernatant was measured at 570 nm on a NanoPhotometer NP80 (Implen). All cell culture conditions were measured in triplicate. To evaluate growth cells were cultured under the intended conditions for the desired time (usually 24 and 48 h), MTT assays were performed and related to the t0 value.

Cell density was evaluated by phase contrast microscopy and quantification of the number of nuclei per area. For the latter, H9c2 cells were seeded in 12 well plates with 5 × 10^4^ cells per well, treated with different ETC inhibitors as desired and nuclei were stained with DAPI (as described below for immunofluorescence). Five random images per well were taken using a 10x objective on a Zeiss Axio Scope A1, nuclei were counted using ImageJ (https://imagej.nih.gov/ij/) and related to the well area covered by the image. Nuclear density from five images was averaged for each well and compared between different treatment conditions.

### siRNA knockdown

Gene knockdown in H9c2 cells was performed using siRNA reagents from Dharmacon/Horizon, including ON-TARGETplus Rat siRNA SMARTpools, siRNA Buffer (B-002000-UB-100), RNase free water (B-003000-WB-100) and DharmaFECT 1 transfection reagent (T-2001). The following siRNA pools were used: Atf4 (L-099212-02), Ddit3 (encoding CHOP, L-088282-02), Hsf1 (L-081010-01), Hspd1 (encoding HSP60, L-092897-02), Nfe2l2 (encoding NRF2, L-080047-02), Nrf1 (L-094657-02), non-target siRNA (D-001810-10). Reconstitution and handling of reagents as well as experimental protocols were performed according to the manufacturer´s instructions. No antibiotics were used in cell culture medium at any time during the procedure. Briefly, H9c2 cells were seeded at a density of 12,500 cells per cm^2^ in 12 well (for MTT assays) or 6 well (for protein isolation) plates. Transfection medium containing 25 nM siRNA and 2 μl/ml transfection reagent was prepared. 24 h after seeding transfection was started by adding 1 ml (12 well plates) or 2 ml (6 well plates) transfection medium containing 10% FBS for 24 h. Subsequently, transfection medium was removed and cells were grown in culture medium containing 10% FBS for another 24 h or 48 h after which MTT assays or protein isolations were performed. All siRNA conditions were run in triplicate and data was replicated in at least two or three independent experiments. Data derived from target gene knockdown were compared to non-target siRNA from the same experiment.

### Immunofluorescence staining

For immunofluorescence staining cells were cultured in 12 well plates on glass coverslips under the intended conditions for the desired time after which the culture medium was aspirated. Cells were washed twice in PBS for 2 min, fixed in 4% PFA in PBS for 10 min and permeabilized in PBS containing 0.25% Triton X-100 for 10 min (all steps at RT). After three washes in PBS for 5 min each blocking was performed in antibody solution (1% BSA, 0.1% Triton X-100, 0.05% Tween 20, 0.05% sodium azide in PBS) containing 5% normal goat serum (Jackson ImmunoResearch) for 1 h at RT, after which cells were incubated over night with primary antibodies at 4°C on a rocking platform. The following primary rabbit antibodies were used: Ki67 (RM-9106, Thermo Fisher, 1:250), phospho-Histone H3 Ser10 (#9701, Cell Signaling Technology, 1:800), ATF4 (#11815, Cell Signaling Technology, 1:200), CHOP (GADD153, sc-575, Santa Cruz, 1:200), cleaved caspase-3 (#9661, Cell Signaling Technology, 1:400). Secondary antibody detection was performed at room temperature for 1 h using an Alexa Fluor 555 conjugated goat anti rabbit secondary antibody (A21428, Thermo Fisher, 1:500). Nuclei were stained with DAPI (#6335, Carl Roth) and glass cover slips were mounted in ProLong Gold antifade reagent (Cell Signaling Technology) on microscopy slides. Imaging was performed using a Zeiss Axio Scope A1 or Axio Observer 7 equipped with the ZEN blue imaging capture software.

### Reactive oxygen species detection

Reactive oxygen species were detected using the fluorescence probes MitoSOX Red (M36008, Thermo Fisher) and CellROX Green (C10444, Thermo Fisher) according to the manufacturer’s instructions. For plate reader detection H9c2 cells were seeded in sterile, black-walled, clear-bottom 96 well microtiter plates (6,000 cells per well) and cultured for 48 h. Cells were subsequently treated with DMSO, Rotenone or Antimycin A for 24 and 48 h. Medium was aspirated and 5 µM MitoSOX in Hank’s Balanced Salt Solution (HBSS) was added in a total volume of 100 µl per well for 15 min at 37°C. Cells were washed three times in HBSS and fluorescence was measured in a CLARIOstar microplate reader (BMG Labtech) with 505 nm excitation and 590 nm emission wavelength. CellROX was added at a concentration of 5 µM in DMEM and incubated for 30 min at 37°C after which cells were washed three times in PBS. Fluorescence was measured with 485 nm excitation and 550 nm emission wavelength. Cells treated as above but without the addition of MitoSOX or CellROX, respectively, were included for autofluorescence correction. To account for differences in cytotoxicity and cell number, MTT assays were performed in the same wells after completion of fluorescence readings. Volumes were adjusted to 96 well formats and MTT was incubated on the cells for 3 h at 37°C. Absorption was measured in a CLARIOstar microplate reader at 570 nm wavelength. For each well MitoSOX and CellROX fluorescence intensity corrected for autofluorescence was normalized to MTT values. Each condition was run in triplicate and the mean value of DMSO treated cells was set as 1.

For fluorescence microscopy of MitoSOX cells were seeded in 8 well chamber slides (94.6140.802, Sarstedt) and treated with DMSO, Rotenone or Antimycin A for 24 h. Medium was aspirated and 5 µM MitoSOX in DMEM was added in a total volume of 500 µl per well for 20 min at 37°C. Cells were washed two times in PBS and imaged.

For the evaluation of mitochondrial membrane potential cells were cultured as above for 1 h or 24 h. Medium was aspirated and 20 nM TMRM (Tetramethylrhodamine Methyl Ester Perchlorate, T668, Thermo Fisher) in DMEM was added in a total volume of 500 µl per well for 30 min at 37°C. Cells were washed two times in PBS and imaged.

### Evaluation of cell size

To measure the surface area of attached H9c2 cells cytoskeletal actin was stained by Alexa Fluor 488 conjugated phalloidin (A12379, Thermo Fisher) to outline cell borders. Cells were cultured in 12 well plates on glass coverslips under the intended conditions for the desired time and subsequently processed as described above for immunofluorescence staining. Cells were incubated with phalloidin (1:40 in PBS) at 4°C over night and nuclei were stained using DAPI. Imaging was performed using a Zeiss Axio Observer 7 equipped with the ZEN blue imaging capture software. Isolated cells in loosely confluent areas of the coverslip with clearly discernable cell borders were selected and cell surface area was measured using ZEN blue software. The mean surface area of 30–50 cells per coverslip was determined with *n* = 4 coverslips per culture condition.

Cell volume was measured in suspension using a Casy cell counter and analyzer (OMNI Life Science). Cells were cultured in 12 well plates under the intended conditions for the desired time and subsequently detached using standard trypsin treatment. Cell suspensions were diluted 1:100 and measured with the following Casy parameters: 3 × 400 µl sample volume, capillary 150 μm, aggregate correction off, size scale 0–30 µm with 0–7.42 µm representing cell debris, 7.43–11.93 µm representing dead cells and 11.94–30 µm representing viable cells. The mean volume of 1,500 up to 4,000 cells was determined per well and averaged from *n* = 4 wells per culture condition.

### Evaluation of cell cycle activity

Bromodeoxyuridine (BrdU, Sigma Aldrich) incorporation was used to assess cell cycle activity in H9c2 and HL-1 cells. Cells were cultured in 12 well plates on glass coverslips under the intended conditions for the desired time and BrdU was added to the culture medium at a concentration of 10 µM for the final 30 min. Cells were washed in PBS and fixed in 70% ethanol for 5 min. DNA was denatured in 2 M HCl for 30 min, cells were washed in PBS and blocked in antibody solution containing 5% normal goat serum (Jackson ImmunoResearch) for 1 h. Cells were incubated with a rat antibody against BrdU (ab6326, 1:1,000, Abcam) at 4°C over night. Secondary detection was performed using an Alexa Fluor 555 conjugated goat anti rat antibody (1:500, Thermo Scientific) for 1 h at room temperature. Nuclei were stained with DAPI and cells were mounted in ProLong Gold antifade reagent (Cell Signaling Technology). Cells were imaged using a Zeiss Axio Scope A1 fluorescence microscope at 20x optical magnification. Five random fields per well/coverslip were imaged, nuclei were counted using the “Find Maxima” function in ImageJ and BrdU signals were counted manually. The percentage of BrdU positive nuclei was calculated per well. Each culture condition or time point was analyzed in triplicates, such that data was averaged from three wells. To further assess proliferation in H9c2 cells immunofluorescence staining for phosphorylated Histone H3 (PHH3) and Ki67 was performed as described above. Proliferation rates were calculated as described for BrdU.

### Evaluation of cell death

Programmed cell death (apoptosis) was detected by TUNEL assays using the ApopTag Fluorescein *In Situ* Apoptosis Detection Kit (S7110, Merck). H9c2 or HL-1 cells were cultured on glas coverslips in 12 well plates under the intended conditions for the desired time. Cells were subsequently washed 2x in PBS and fixed in 1% PFA for 15 min at room temperature. Positive controls were treated with DNase (RNase-free DNase set, Qiagen) for 10 min. Subsequent procedures were then performed according to the manufacturer´s instructions. In addition, apoptosis was detected by immunofluorescence staining for cleaved caspase-3 as described above. Nuclei were stained with DAPI and cells were mounted in ProLong Gold antifade reagent (Cell Signaling Technology). Cells were imaged using a Zeiss Axio Scope A1 fluorescence microscope at 20x optical magnification. Five to ten random fields per well/coverslip were imaged, nuclei were counted using the “Find Maxima” function in ImageJ and TUNEL or cleaved caspase-3 signals were counted manually. The percentage of apoptotic nuclei was calculated per well and for each culture condition or time point data was averaged.

To determine cellular disintegration and necrosis in HL-1 cells the release of lactate dehydrogenase into the culture medium was measured using the LDH Cytotoxicity Assay Kit from Cayman Chemical (#601170). 10^5^ cells were seeded in 12 well plates for 24 h followed by Antimycin A and Rotenone treatment with equivalent volumes of DMSO serving as control. Culture medium was collected 24 and 48 h after initiation of treatment. 100 µl of cell culture supernatant was used for the assay which was performed in white opaque 96 well flat bottom microplates according to the manufacturer´s instructions. Absorbance at 490 nm was measured in a CLARIOstar microplate reader (BMG Labtech) after 90 min. Three different wells per condition were analyzed and measurements were performed in duplicate for each well. Cell culture medium not in contact with any cells was included in the assay such that its absorbance was subtracted for blank correction.

### Caspase-3 activity assay

H9c2 cells were seeded in 6 well plates (2 × 10^5^ cells per well) and treated with Rotenone or Antimycin A for 24 h. Protein lysates were prepared as described below for Western Blots. 10 µl lysate were mixed with 90 µl reaction solution [10 mM HEPES pH = 7, 40 mM β-glycerophosphate, 50 mM NaCl, 2 mM MgCl_2_, 5 mM EGTA, 100 μg/ml BSA, 0.1% CHAPS, 11 µM Ac-DEVD-AMC (14986, Cayman Chemical)] in black-walled 96 well plates. Each sample was measured in triplicate. Plates were incubated at 37°C in a CLARIOstar microplate reader (BMG Labtech) and fluorescence was measured immediately (t0) as well as after 30 and 60 min with 350 nm excitation and 450 nm emission wavelength. The increase in fluorescence intensity per well (Δ t0 versus 60 min) was used to indicate caspase-3 activity and the values were normalized to the protein concentration of the respective sample. Triplicate measurements were averaged for each well and the mean value of the DMSO treated wells was set as 1.

### Cytometry for Annexin V and 7-AAD staining

To detect cell death in cytometry the PE Annexin V Apoptosis Detection Kit I (559763, BD Pharmingen) was used. 1.5 × 10^5^ H9c2 cells were seeded on 6 well plates and treated with DMSO, Rotenone or Antimycin A for 24 h. Cells were washed with PBS and carefully detached using 500 µl TrypLE Select reagent (A1285901, Thermo Fisher) per well for 5 min at 37°C. Cells were resuspended in 500 µl culture medium, transferred to 5 ml cytometry tubes and centrifuged at 1,500 rpm for 5 min. The supernatant was discarded, cells were washed twice in 1 ml cold PBS and resuspended in 100 µl binding buffer. 3 μl PE Annexin V and 4 µl 7-AAD (7-aminoactinomycin D) were added, the suspension was mixed by gentle vortexing and incubated for 15 min at room temperature in the dark before adding 250 µl binding buffer. Cells were analyzed within 1 h using a BD FACSLyric flow cytometer (12-color, BD Biosciences). Flow cytometry data was analyzed using FlowJo software (version 10.8.1, BD Biosciences). Cell debris was filtered out based on forward and side scatter and cells were gated to identify viable (Annexin V/7-AAD double negative), early apoptotic (Annexin V positive/7-AAD negative), late apoptotic (Annexin V/7-AAD double positive) and dead (7-AAD positive) cells. The assay was performed in 4 wells per treatment and replicated in two independent experiments. Percentages of the different cellular states were determined for each well (based on 17,000 to 19,000 analyzed cells per well) and averaged for the respective treatment group.

### Western Blot analyses

For protein isolation H9c2 and HL-1 cells were grown in 6 well plates and treated as desired. Cells were washed in PBS and lysed in 50 μl cell lysis buffer (#9803, Cell Signaling Technology) supplemented with protease (Complete Protease Inhibitor Cocktail Tablets, Roche Diagnostics) and phosphatase (PhosSTOP Phosphatase Inhibitor Cocktail Tablets, Roche) inhibitors for 10 min on ice. Cells were mechanically disrupted using cell scrapers, the lysate was transferred to a reaction tube and centrifuged at 12,000 × g for 10 min at 4°C. Protein concentration in the supernatant was determined by a modified Lowry assay (DC Protein Assay, BIO-RAD). For samples on the same gel equal protein amounts (usually 20 μg) were loaded, separated using denaturing polyacrylamide gel electrophoresis (SDS-PAGE) and blotted onto nitrocellulose (GE Healthcare) or PVDF (Millipore) membranes. Membranes were blocked for 1 h in 5% non-fat dry milk (Carl Roth) in TBS-T and incubated with primary antibodies at 4°C over night. A list of primary antibodies used in this study is provided in Supplementary Table S1. Antibodies against GAPDH, α-Tubulin and Vinculin were used for loading control and for normalization upon densitometric quantifications. Secondary detection was performed using horseradish peroxidase (HRP)-linked secondary antibodies (Cell Signaling Technology, #7074 and #7076, 1:2,000). Standard enhanced chemiluminescence (ECL) reaction was used for abundant proteins whereas weakly expressed proteins were detected using the SuperSignal West Femto Maximum Sensitivity Substrate (Thermo Scientific). ECL signals were imaged with the ChemiDoc XRS+ (BIO-RAD) system. Intensity of detected protein bands was quantified by densitometry using ImageJ (https://imagej.nih.gov/ij/) or Image Lab (BIO-RAD) software. Western blot membranes were usually cut horizontally based on the visible bands of the molecular size marker to allow simultaneous detection of multiple proteins of different molecular weight. As a result, the same loading control serves for normalization of various proteins that were run on the same gel and detected on the same membrane. Therefore, identical blot images for loading controls might appear in different figure panels showing western blot data.

### Analyses of RNA expression by qRT-PCR

Total RNA from H9c2 and HL-1 cells was isolated using the RNeasy Kit (Qiagen). Cells were grown in 6 well plates under the intended conditions for the desired time, washed in PBS and lysed using RLT buffer supplemented with β-mercaptoethanol. The lysate was centrifuged to remove cell debris, the supernatant was loaded on RNeasy spin columns and RNA was purified according to the manufacturer´s instructions including digestion of genomic DNA on the column (RNase-free DNase set, Qiagen).

Isolated cellular RNA was reverse transcribed using M-MuLV reverse transcriptase (New England BioLabs) and random hexamer primers. Quantitative real time PCR was performed using the SsoAdvanced Universal SYBR Green Supermix (BIO-RAD) on the CFX Connect Real-Time PCR Detection System (BIO-RAD). Primers were obtained from BioTeZ (Berlin, Germany) or Eurofins (Ebersberg, Germany) and sequences are provided in Supplementary Table S2. All primers and PCR conditions were optimized to PCR efficiencies between 90 and 110% and a correlation coefficient ≥0.990 using cDNA dilution series. All samples were analyzed in triplicates and normalized to Polr2a (RNA polymerase II subunit A) expression. Relative expression differences between groups were determined using the ΔΔCT method.

### Statistical analyses

All data were analyzed with SPSS (IBM) or Excel 2010 (Microsoft) software and are presented as mean ± standard error of the mean (SEM). Data sets were tested for normal distribution by Shapiro-Wilk test and homogeneity of variances between groups was assessed by Levene’s test. If these criteria were met, differences between two groups were evaluated with unpaired, two-sided student’s *t*-test and those among multiple groups with one-way analysis of variance (ANOVA) followed by Bonferroni *post-hoc* test. For multiple groups with unequal variance Games Howell *post-hoc* test was performed. Differences between multiple groups with non-normal distribution were evaluated with non-parametric Kruskal Wallis one-way analysis of variance followed by pairwise comparison of groups. A probability (*p*) value less than 0.05 was considered to indicate statistical significance (*p < 0.05, **p < 0.01 and ***p < 0.001).

## Results

### H9c2 cells tolerate inhibition of mitochondrial complex III but not complex I or V

**FIGURE 1:**
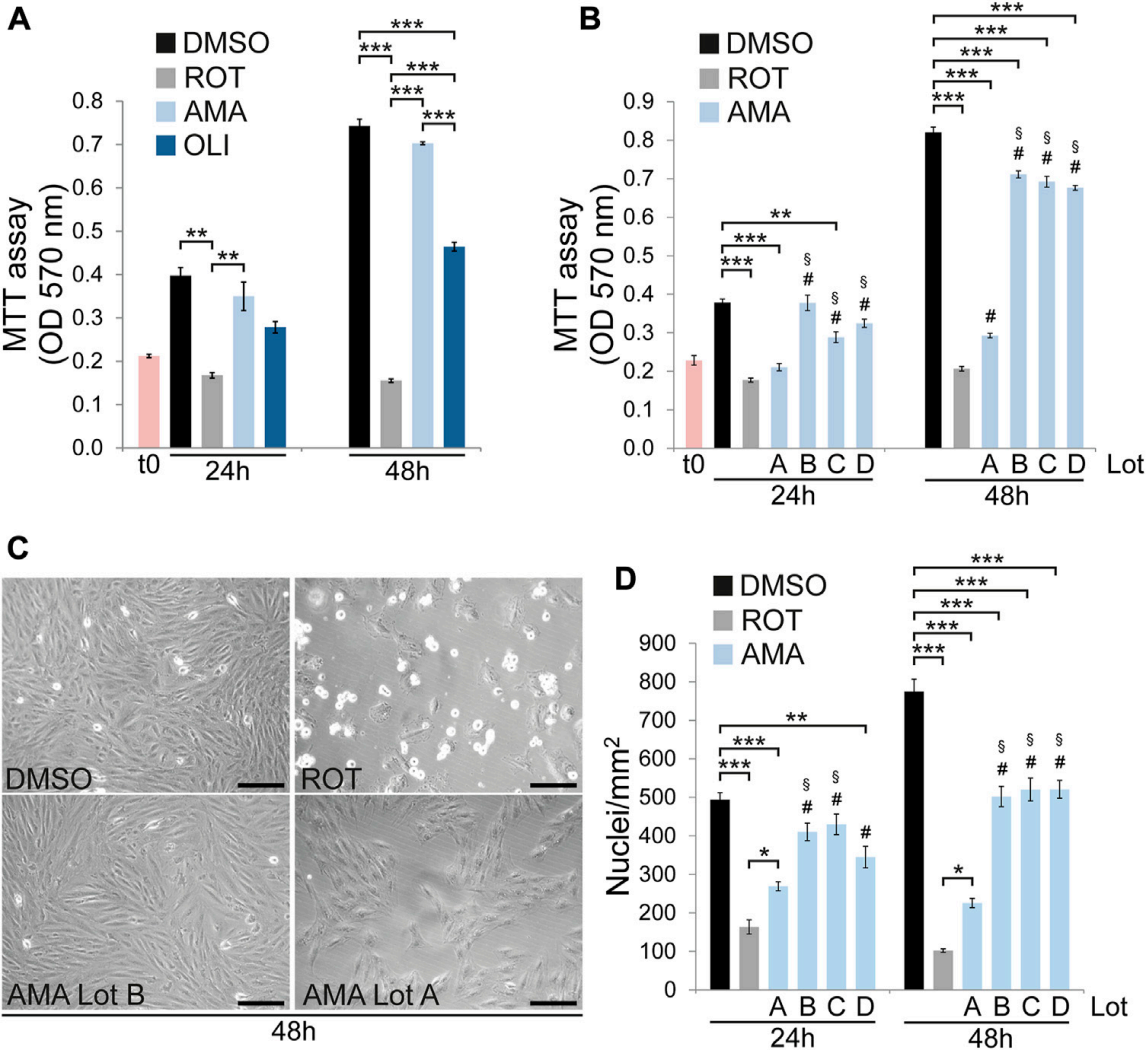
H9c2 cells partially tolerate mitochondrial complex III but not I or V inhibition. **(A)** MTT assays of H9c2 cells treated with DMSO (included as control), rotenone (ROT, 10 µM), antimycin A (AMA, 50 µM) and oligomycin (OLI, 5 µM) for 24 and 48 h. **(B)** MTT assays of H9c2 cells treated with ROT (10 µM) and four different lots of AMA (50 µM each) for 24 and 48 h. **(C)** Phase contrast microscopy images of H9c2 cells treated with DMSO, ROT and AMA lots A and B [same as in (B)] for 48 h (scale bar = 200 µm). **(D)** Nuclear density of H9c2 cells treated with ROT and four different lots of AMA [same as in (B)] for 24 and 48 h. (*n* = 4 wells per treatment in **(A)**, **(B)** and **(D)**, *p < 0.05, **p < 0.01, ***p < 0.001, #*p* < 0.001 versus ROT, §*p* < 0.001 versus AMA Lot A. t0 in **(A)** and **(B)** represents MTT values at the onset of treatment).

To investigate survival and growth of H9c2 cells in response to mitochondrial dysfunction cells were treated with the complex I inhibitor rotenone (ROT), the complex III inhibitor antimycin A (AMA) and the complex V inhibitor oligomycin (OLI). MTT assays were performed to determine cell growth and cytotoxicity after 24 and 48 h. Whereas different concentrations of AMA are well tolerated, ROT and OLI significantly reduce cell growth and survival compared to DMSO treated controls (Figure 1A; Supplementary Figure S1). These results correlated well with phase contrast microscopy where ROT clearly reduces cell density after 24 and 48 h whereas AMA treated cells were indistinguishable from DMSO at both time points (Figure 1C). Unless noted otherwise the following final concentrations of electron transport chain (ETC) inhibitors were used for all cell treatments throughout the study: 50 μM AMA, 10 µM ROT and 5 µM OLI. Surprisingly, throughout the course of the project we observed one particular AMA lot which significantly reduces H9c2 cell growth to levels similar to ROT. We therefore repeated the experiments using four different AMA lots from three different suppliers. These data verified that ROT and only one AMA lot clearly reduce MTT values after 24 and 48 h whereas three AMA lots had either no or rather mild effects compared to DMSO (Figures 1B,C). To characterize the molecular and cellular effects caused by that particular AMA lot compared to the others we included it in some downstream experiments. Importantly, however, unless this “growth inhibitory” AMA (hereafter referred to as *gi*AMA) is specifically mentioned, all other experiments throughout the study were performed with AMA exhibiting no or mild effects in MTT results.

To identify potential factors influencing H9c2 cell growth upon AMA treatment, cell culture conditions were thoroughly characterized. Cells can adapt to mitochondrial dysfunction by switching to glycolysis. In fact, immature cardiomyocytes in the embryonic and fetal heart primarily rely on aerobic glycolysis and only switch to an oxidative metabolism predominantly fueled by fatty acids after birth (Garbern and Lee, 2021). To test whether H9c2 cells rely on glucose availability to grow and survive upon mitochondrial dysfunction we reduced glucose concentration in the culture medium. However, low glucose conditions do not negatively affect growth of H9c2 cells upon AMA treatment over a period of 48 h (Supplementary Figure S2A). It has been reported that passaging has an effect on cellular stress response with late passages exhibiting an increased susceptibility to doxorubicin compared to early passages (Witek et al., 2016). H9c2 cells in late passages generally tend to grow slower over a period of 48 h but we did not observe a passage dependent effect of AMA on growth and survival when compared to DMSO (Supplementary Figure S2B). Furthermore, cell culture medium was supplemented with three different FBS (fetal bovine serum) batches. Surprisingly, whereas two of them confirmed the previous results showing no effect of AMA on H9c2 cell growth, with one FBS batch H9c2 cell density after 48 h was reduced (Supplementary Figure S2C). However, this effect was still not as severe as detected for ROT. In conclusion, whereas ROT and OLI reduce growth and survival of H9c2 cells mitochondrial complex III inhibition by AMA is well tolerated. However, the latter is influenced by AMA composition as well as potential cytoprotective factors contained in some but not all FBS batches. All subsequent experiments were therefore performed with FBS that did not impair growth and survival of AMA treated H9c2 cells.

### Antimycin A treatment does not impair proliferation of H9c2 cells but moderately induces cell death

MTT assay is an indirect estimation of cell growth and cytotoxicity which relies on cellular redox metabolism as a measure of cell number or cell mass. We therefore quantified cell density in ROT and AMA treated H9c2 cells based on DAPI stained nuclei in fluorescence microscopy. In accordance with MTT data, ROT dramatically reduces the number of nuclei per area after 24 and 48 h. In contrast, AMA had no effect after 24 h but mildly reduces nuclear density after 48 h compared to DMSO (Figure 1D). The *gi*AMA (Lot A) had an intermediate effect ranging between ROT and AMA. These data show that mitochondrial complex I inhibition by ROT severely reduces the number of H9c2 cells after 24 and 48 h whereas complex III inhibition by AMA is well tolerated over the first 24 h and only causes mild effects after 48 h.

**FIGURE 2:**
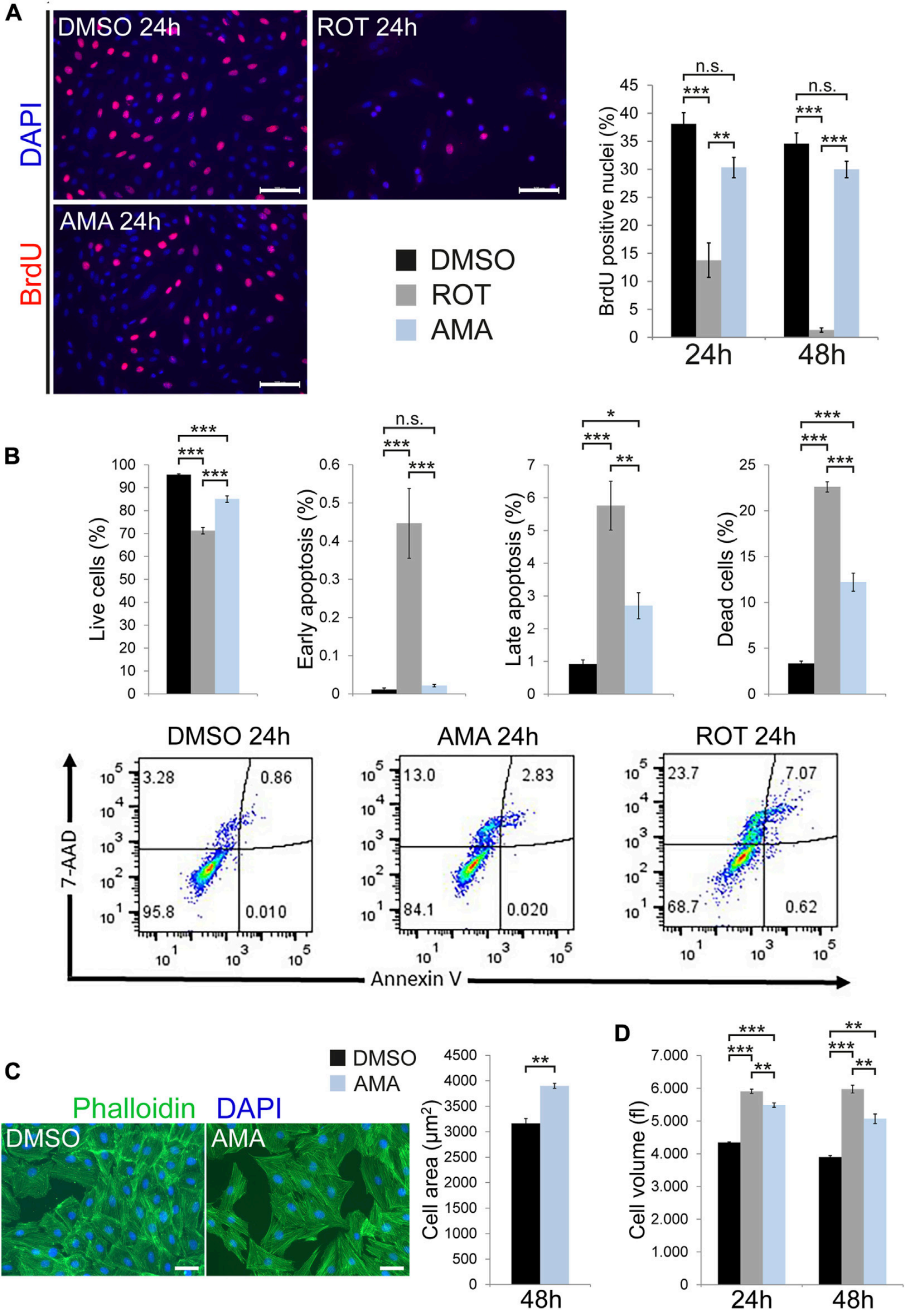
Apoptosis, cell cycle activity and cell size in H9c2 cells treated with inhibitors of mitochondrial respiration. **(A)** Fluorescence microscopy images showing BrdU incorporation in H9c2 cells treated with DMSO, AMA (50 µM) or ROT (10 µM) for 24 h. Cell cycle activity was evaluated as the number of BrdU positive nuclei (stained in red) related to the overall number of nuclei (stained in blue using DAPI, *n* = 3 wells per treatment, scale bar = 100 µm). **(B)** Cell death was determined in DMSO, AMA and ROT treated H9c2 cells after 24 h using Annexin V and 7-AAD staining in cytometry. Annexin V positive but 7-AAD negative cells were considered to represent early apoptosis whereas double positive cells represent late apoptosis. 7-AAD positive but Annexin V negative cells are considered dead and double negative cells are alive (*n* = 4 wells per treatment). **(C)** H9c2 cells were treated with DMSO and AMA for 48 h. Cell surface area was measured in fluorescence microscopy images based on phalloidin staining of actin filaments (in green) to outline cell borders (*n* = 4 wells per treatment; nuclei are stained in blue using DAPI; scale bar = 50 µm). **(D)** H9c2 cells were treated with DMSO, ROT and AMA for 24 and 48 h. Cell volume was evaluated by flow cytometry (*n* = 3 wells per treatment). (*p < 0.05, **p < 0.01, ***p < 0.001, n.s. = non-significant).

A reduction in cell number can be caused by impaired proliferation or induction of cell death. Cell cycle activity was assessed by BrdU incorporation, which showed a clear reduction in ROT treated H9c2 cells compared to DMSO after 24 and 48 h. In contrast, AMA had no effect on the percentage of BrdU positive nuclei at both time points (Figure 2A). To further confirm these data we determined cell cycle activity by Ki67 immunostaining (Supplementary Figure S3A) and quantified the number of mitotic cells by immunofluorescence detection of phosphorylated histone H3 (PHH3) (Supplementary Figure S3B). These analyses revealed that AMA has no major effect on cell cycle activity and mitosis of H9c2 cells compared to DMSO after 6, 24 and 48 h. We determined programmed cell death (apoptosis) *via* cleavage of caspase-3 in Western Blot and immunofluorescence analyses. Both methods consistently showed that ROT induces apoptosis in H9c2 cells compared to DMSO whereas AMA does not (Supplementary Figures S4A,B). This data was furthermore confirmed by TUNEL assay (Supplementary Figure S4C). In addition, we monitored caspase-3 activation by applying the fluorescent substrate Ac-DEVD-AMC to lysates of H9c2 cells treated with DMSO, ROT and AMA. These data showed strong caspase-3 activation by ROT but not AMA (Supplementary Figure S4D). To also consider caspase independent cell death mechanisms and necrosis we applied Annexin V and 7-AAD staining in cytometry. These data confirmed a robust induction of cell death in H9c2 cells by ROT whereas AMA had a much milder effect compared to DMSO after 24 h (Figure 2B). Interestingly, only later stages of apoptosis (represented by Annexin V and 7-AAD double positive cells) were induced by AMA whereas early stages (represented by Annexin V positive but 7-AAD negative cells) were not (Figure 2B).

Finally, MTT assays primarily detect cell mass such that a reduction in cell number could be compensated by increased cell size. In addition, cellular stress can lead to hypertrophic growth in cardiomyocytes. Indeed, AMA increases the surface area in H9c2 cells compared to DMSO (Figure 2C). Furthermore, cell volume determined by cytometry was increased by both ROT and AMA (Figure 2D). These results are in agreement with ROS and oxidative stress inducing cardiomyocyte hypertrophy (Sag et al., 2014).

In conclusion, mitochondrial complex I inhibition by ROT induces cell cycle arrest and caspase-3 mediated apoptosis in H9c2 cells whereas complex III inhibition by AMA does not affect proliferation but moderately causes caspase independent cell death.

### Mitochondrial dysfunction induces various stress response pathways in H9c2 cells

**FIGURE 3:**
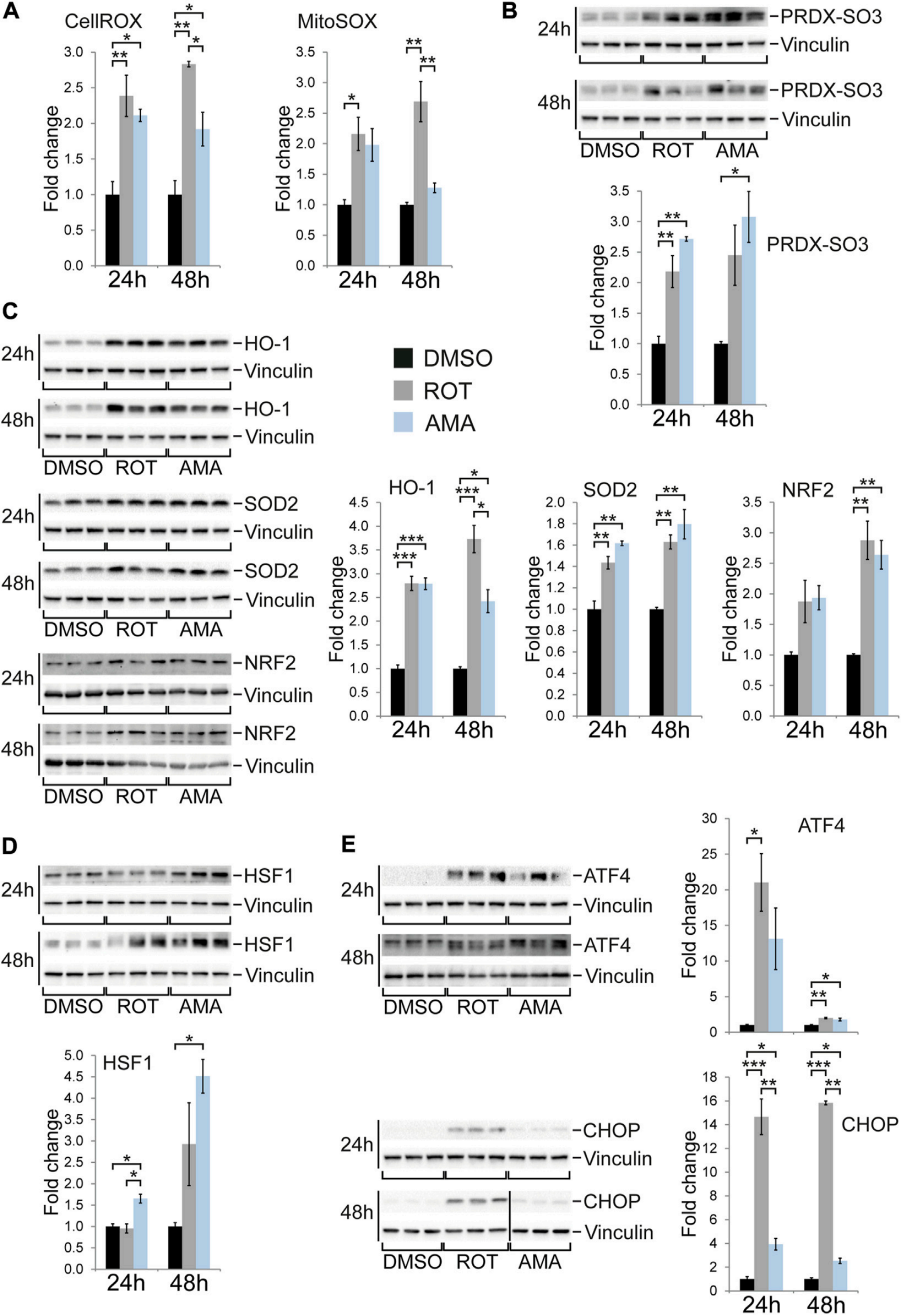
Oxidative stress and cellular stress response in H9c2 cells treated with rotenone and antimycin A. **(A)** Generation of reactive oxygen species was evaluated using the fluorescence probes CellROX and MitoSOX in H9c2 cells treated with ROT (10 µM) and AMA (50 µM) for 24 and 48 h. Fluorescence intensity was measured in a plate reader and related to DMSO treated controls. **(B)** Western blots for hyperoxidized peroxiredoxin (PRDX-SO3) in H9c2 cells treated with ROT and AMA for 24 and 48 h. **(C)** Western blots of enzymes involved in antioxidative defense [heme oxygenase 1 (HO-1) and superoxide dismutase 2 (SOD2)] as well as the oxidative stress responsive transcription factor NRF2 in H9c2 cells treated with ROT and AMA for 24 and 48 h. **(D)** Western blots of HSF1, representing a key transcription factor of the heat shock response, in H9c2 cells treated with ROT and AMA for 24 and 48 h. **(E)** Western blots of ATF4 and CHOP, representing key transcription factors of the integrated stress response, in H9c2 cells treated with ROT and AMA for 24 and 48 h. The vertical black line indicates that samples were run on the same gel but were non-contiguous. [*p < 0.05, **p < 0.01, ***p < 0.001, *n* = 3 wells per treatment in **(A**–**E)**].

Given the different outcome of mitochondrial complex I compared to complex III inhibition in terms of cell death and proliferation of H9c2 cells, we sought to identify the underlying molecular stress response mechanisms. We first quantified reactive oxygen species (ROS) in the cell and specifically within mitochondria using fluorescence based assays as well as microscopy (Mukhopadhyay et al., 2007). ROT and AMA both increased ROS generation to similar levels after 24 h when compared to DMSO (Figure 3A; Supplementary Figure S5A). Hyperoxidation of specific cysteines within peroxiredoxin (PRDX) proteins can result in sulfonic acid (SO3H) residues indicating oxidative stress. Western blot experiments revealed that ROT and AMA increased PRDX-SO3 to similar levels in H9c2 cells when compared to DMSO (Figure 3B). Mitochondrial integrity can be detected by TMRM, a red fluorescent dye which integrates into membranes of intact mitochondria but is lost upon membrane depolarization. Fluorescent microscopy revealed that both ROT and AMA reduce TMRM staining in H9c2 cells after 1 h compared to DMSO (Supplementary Figure S5B). In summary, these data indicate that oxidative stress and mitochondrial impairment is similar in H9c2 cells upon ROT or AMA treatment.

Next we tested the activation of molecular stress pathways in ROT and AMA treated H9c2 cells. Mitochondrial dysfunction can induce molecular mechanisms to attenuate oxidative stress and embryonic cardiomyocytes induce SOD2 upon complex III inhibition *in vivo* (Magarin et al., 2016). Indeed, various enzymes and proteins involved in ROS detoxification such as HO-1, SOD1, SOD2, and PRDX3 where induced (Figure 3C; Supplementary Figure S6B) whereas TRX2 shows unaltered or reduced expression in ROT and AMA treated H9c2 cells (Supplementary Figure S6B). In agreement, NRF2, a key transcription factor regulating genes involved in antioxidative defense (Chen and Maltagliati, 2018), was induced by both ETC inhibitors (Figure 3C).

Cardiomyocytes in the embryonic mouse heart have been shown to induce the mitochondrial chaperone HSP60 (Magarin et al., 2016), a component of the UPR^mt^ (Shpilka and Haynes, 2018) as well as the heat shock response (HSR) (Gomez-Pastor et al., 2018). Western blot analyses revealed increased expression of the key HSR transcription factor HSF1 in AMA treated H9c2 cells (Figure 3D). HSP90 and HSP60 expression was unaffected whereas HSP70 was downregulated by ROT but not AMA when compared to DMSO (Supplementary Figure S6A).

Cellular stress can be relayed to the stress activated protein kinases p38 and JNK (c-Jun N-terminal kinase) (Wang, 2007). Whereas phosphorylation of JNK is only mildly affected by ROT and AMA in H9c2 cells, ROT appears to reduce total JNK protein levels (Supplementary Figure S6C). Importantly, phosphorylation of p38 MAP kinase is induced by both ETC inhibitors after 24 h but by ROT only after 48 h (Supplementary Figure S6C).

*In vivo* studies have shown that mitochondrial complex III inhibition activates the unfolded protein response (UPR) (Shpilka and Haynes, 2018) and integrated stress response (ISR) (Pakos-Zebrucka et al., 2016) in mouse embryonic cardiomyocytes (Magarin et al., 2016). Consistently, western blot and immunofluorescence analyses showed that ROT and AMA induce the ISR transcription factors ATF4 and CHOP (also known as GADD153) in H9c2 cells after 24 and 48 h (Figure 3E; Supplementary Figures S7A,B). Whereas the induction of ATF4 was similar, ROT had a much stronger effect on CHOP expression compared to AMA after 48 h (Figure 3E). Interestingly, phosphorylation of eIF2α, which has been reported to precede the stress induced induction of ATF4 and CHOP (Pakos-Zebrucka et al., 2016), was not detected in AMA treated H9c2 cells, which could be due to increased expression of the eIF2α phosphatase GADD34 (Supplementary Figure S7C).

In summary, ROT and AMA both induce components of different cellular stress response mechanisms in H9c2 cells, including the ISR, UPR, HSR, antioxidative defense, and stress kinases. The most striking differences between complex I and III inhibition seems to be a prolonged induction of CHOP and p38 phosphorylation by ROT compared to AMA.

### Antimycin A with growth inhibitory effects induces a markedly increased cellular stress level

As described above, we identified one particular AMA lot (*gi*AMA or lot A in Figure 1) which reduces MTT values in H9c2 cells whereas three other lots do not. Interestingly, *gi*AMA induces cell cycle arrest (as revealed by BrdU incorporation, Supplementary Figure S3C) but not apoptosis (Supplementary Figures S4A–C). Western blot analyses revealed that *gi*AMA caused a much higher induction of CHOP and ATF4 as compared to other AMA lots (Supplementary Figure S8A). Similarly, induction of HO-1 was markedly higher in *gi*AMA treated cells (Supplementary Figure S8A), whereas induction of HSF1 was similar in all AMA lots. Interestingly, hyperoxidation of PRDX (i.e., PRDX-SO3) was induced to similar levels by all AMA lots compared to DMSO (Supplementary Figure S8A). These data suggest that whereas all tested AMA lots induce expression of intracellular stress markers, *gi*AMA increases some of the latter to much higher levels. In particular, the excessive induction of ATF4 and CHOP might explain the negative effect of *gi*AMA on H9c2 cell proliferation, whereas the level of oxidative stress appears to be similar in all AMA lots.

We observed that OLI had an inhibitory effect on H9c2 cell growth that was somewhat intermediate between ROT and AMA (Figure 1A). Western blot analyses revealed that OLI increases expression of ATF4, CHOP and HO-1 as well as phosphorylation of p38 to higher levels when compared to AMA (Supplementary Figure S8B). In summary, different inhibitors of mitochondrial respiratory chain complexes have variable effects on H9c2 cell survival and growth, which seem to correlate with the level and signature of intracellular stress induction.

### Baseline expression of stress related proteins is different in H9c2 versus HL-1 cells

**FIGURE 4:**
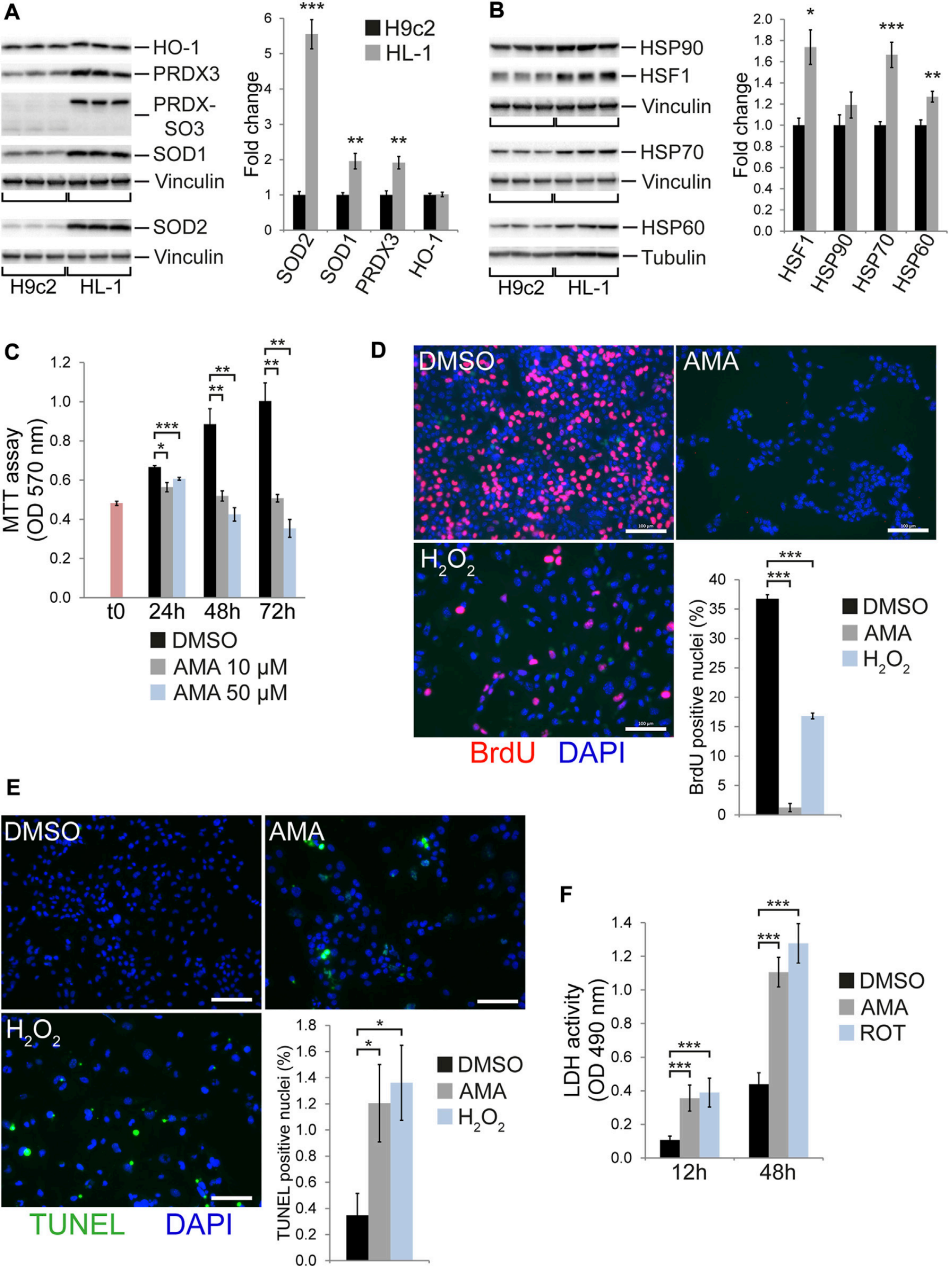
HL-1 cells undergo cell death and cell cycle arrest in response to antimycin A treatment. Western blots showing baseline expression of **(A)** antioxidative proteins and enzymes as well as hyperoxidized peroxiredoxin (PRDX-SO3) and **(B)** HSF1 and chaperones (heat shock proteins) in H9c2 compared to HL-1 cells (*n* = 6 wells per group). **(C)** MTT assays of HL-1 cells treated with DMSO and 10 µM or 50 µM AMA for 24, 48 and 72 h (*n* = 3 wells per treatment, t0 represents MTT values at the onset of treatment). **(D)** Fluorescence microscopy images showing BrdU incorporation in HL-1 cells treated with DMSO, AMA (50 µM) or H_2_O_2_ (500 µM) for 48 h. Cell cycle activity was evaluated as the number of BrdU positive nuclei (stained in red) related to the overall number of nuclei (stained in blue using DAPI, *n* = 3 wells per treatment, scale bar = 100 µm). **(E)** Fluorescence microscopy images showing TUNEL staining in HL-1 cells treated with DMSO, AMA (50 µM) or H_2_O_2_ (500 µM) for 48 h. Apoptosis rates were evaluated as the number of TUNEL positive nuclei (stained in green) related to the overall number of nuclei (stained in blue using DAPI, *n* = 5 wells per treatment, scale bar = 100 µm). **(F)** LDH activity was measured in the culture medium of HL-1 cells treated with AMA (50 µM) or ROT (10 µM) for 12 and 48 h (*n* = 3 wells per treatment). (*p < 0.05, **p < 0.01, ***p < 0.001)

H9c2 cells represent weakly differentiated non-contractile cardiomyoblasts derived from the embryonic rat heart (Kimes and Brandt, 1976; Hescheler et al., 1991) whereas HL-1 is a contractile cell line derived from an adult murine atrial tumor (Claycomb et al., 1998). Cardiomyocyte differentiation *in vivo* includes metabolic reprogramming from glycolysis in the prenatal heart towards an oxidative metabolism after birth (Garbern and Lee, 2021). The latter comes with elevated levels of oxidative stress due to increased ROS generation along the mitochondrial respiratory chain. Consistently, HL-1 cells show higher levels of PRDX hyperoxidation (PRDX-SO3) and express markedly higher levels of proteins and enzymes involved in redox homeostasis, (such as SOD1, SOD2 or PRDX3) when compared to H9c2 cells (Figure 4A). Interestingly, HO-1 expression was not different in H9c2 versus HL-1 cells (Figure 4A). HL-1 cells furthermore express higher levels of HSF1 as well as the chaperones HSP70 and HSP60 (but not HSP90) (Figure 4B). In contrast, phosphorylation of the stress kinases JNK and p38 is reduced in HL-1 compared to H9c2 cells (Supplementary Figure S9A). When investigating the ISR/UPR we observed similar levels of eIF2α phosphorylation between the cell lines but increased expression of the eIF2α phosphatase GADD34 as well as the mitochondrial protease and UPR^mt^ component ClpP in HL-1 compared to H9c2 cells (Supplementary Figure S9B). Strikingly, ATF4 and CHOP expression was much lower in HL-1 compared to H9c2 cells (Supplementary Figure S8B). These data indicate remarkable differences in baseline expression of proteins involved in cellular stress response, oxidative defense and protein homeostasis between HL-1 and H9c2 cells, some of which are in agreement with advanced differentiation.

### HL-1 cells are highly susceptible to antimycin A induced cell death and growth arrest

To test whether tolerance towards mitochondrial complex III inhibition changes with the degree of differentiation we treated HL-1 cells with AMA. The latter causes growth arrest and cell loss at AMA concentrations much lower (10 µM) as compared to those tolerated by H9c2 cells (50 µM) (Figure 4C). This is due to an almost complete inhibition of cell cycle activity (Figure 4D) in combination with the induction of apoptosis (Figure 4E) and cellular disintegration (Figure 4F). Similar to H9c2 cells, HL-1 cells induce protein expression of ATF4 and CHOP upon AMA treatment (Supplementary Figure S10A). In agreement, RNA expression of the ATF4 target genes *Ddit3* (encoding CHOP), *Asns* and *Trib3* was increased by AMA (Supplementary Figure S10B). In contrast, HSF1 and proteins involved in UPR^mt^ (such as HSP60, ClpP and PMPCB) were not differentially expressed in AMA compared to DMSO treated HL-1 cells (Supplementary Figure S10C), whereas AMA increases phosphorylation of p38 MAP kinase (Supplementary Figure S10D). These data reveal that contractile cells resembling differentiated cardiomyocytes are highly susceptible to mitochondrial complex III inhibition and rapidly undergo cell cycle arrest and death.

### The integrated stress response is not required for growth and survival of H9c2 cells upon antimycin A treatment

**FIGURE 5:**
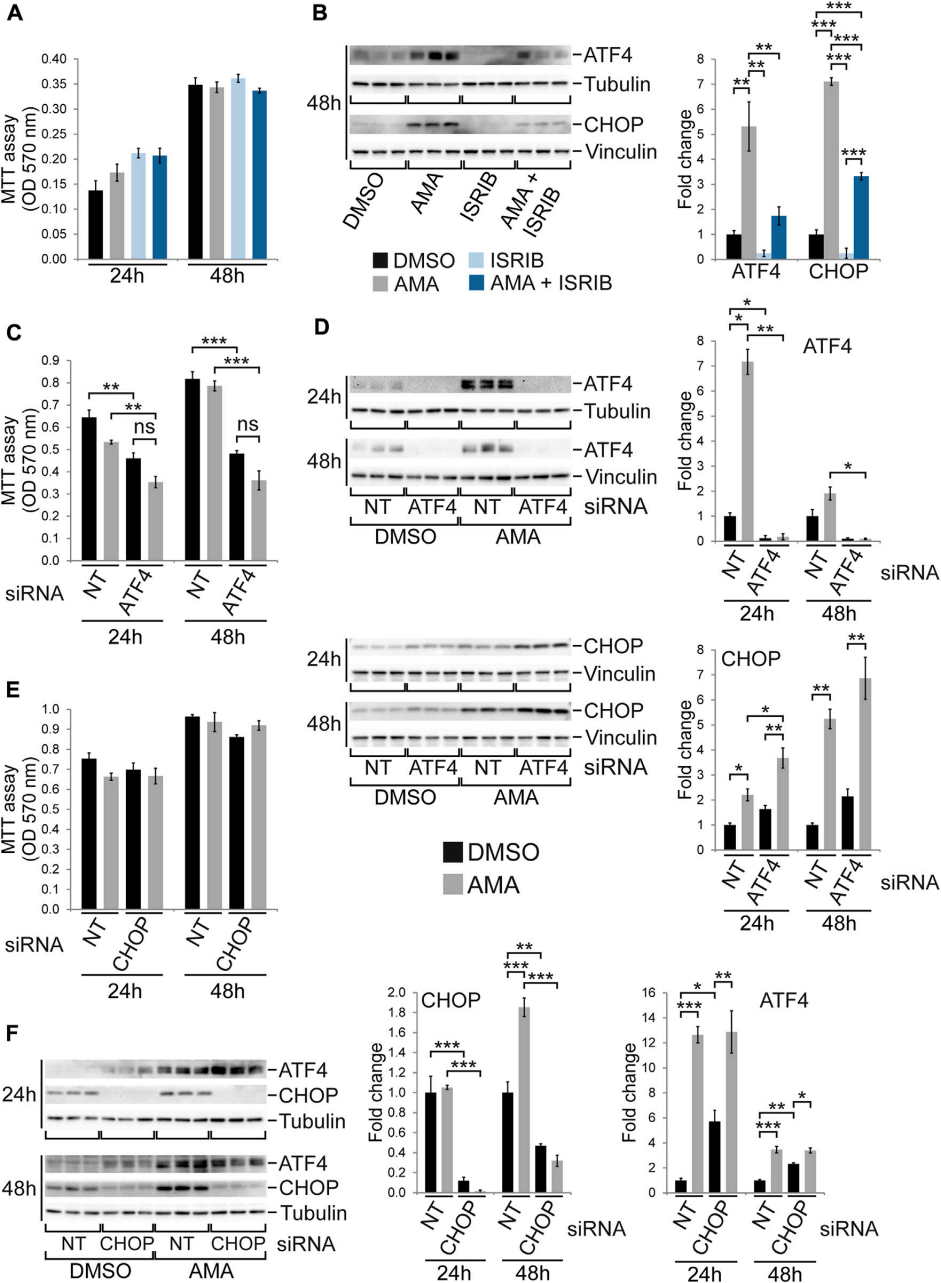
The ISR is not required for growth and survival of H9c2 cells upon antimycin A treatment. **(A)** MTT assays of H9c2 cells treated with DMSO, AMA (50 µM), ISRIB (200 nM) or a combination of AMA and ISRIB for 24 and 48 h. **(B)** Western blots showing protein expression of the ISR components ATF4 and CHOP after 48 h treatment as outlined in **(A)** [*n* = 3 wells per treatment in **(A)** and **(B)**]. **(C)** MTT assays of H9c2 cells transfected with non-target (NT) or ATF4 siRNA and subsequently treated with DMSO or AMA for 24 and 48 h. **(D)** Western blots showing protein expression of the ISR components ATF4 and CHOP in H9c2 cells treated as outlined in **(C)** (*n* = 3 wells per treatment in **(C)** and **(D)**). **(E)** MTT assays of H9c2 cells transfected with non-target (NT) or CHOP siRNA and subsequently treated with DMSO or AMA for 24 and 48 h. **(F)** Western blots showing protein expression of the ISR components ATF4 and CHOP in H9c2 cells treated as outlined in **(E)** [*n* = 3 wells per treatment in **(E)** and **(F)**]. (*p < 0.05, **p < 0.01, ***p < 0.001, n.s. = non-significant).

Given the robust induction of the ISR in H9c2 as well as HL-1 cells upon AMA treatment but the much higher baseline expression of ATF4 and CHOP in H9c2 cells (Supplementary Figure S9B) we speculated that the ISR might protect H9c2 cells from mitochondrial complex III inhibition. The small molecule ISRIB has been reported to inhibit the ISR downstream of eIF2α phosphorylation (Sidrauski et al., 2015) and by itself does not affect H9c2 cell growth (Supplementary Figure S11A). Preincubation of H9c2 cells with ISRIB followed by co-treatment with DMSO or AMA for up to 48 h reduces ATF4 and CHOP protein levels in DMSO treated cells and, more importantly, prevents the induction of ATF4 while attenuating the induction of CHOP by AMA (Figure 5B). However, ISRIB does not affect growth of H9c2 cells upon AMA treatment for up to 48 h (Figure 5A). To further confirm these findings we silenced ATF4 and CHOP by siRNA in H9c2 cells followed by AMA treatment. The knockdown of ATF4 was highly efficient and completely prevented its RNA and protein induction by AMA (Figure 5D; Supplementary Figure S11B) as well as increased RNA expression of the ATF4 target genes *Asns* and *Trib3* (Supplementary Figure S11B). Interestingly, RNA expression of *Ddit3* (encoding CHOP) was unaffected by ATF4 silencing and CHOP protein levels were slightly increased in DMSO and AMA treated H9c2 cells after ATF4 knockdown (Figure 5D; Supplementary Figure S11B). Most importantly, silencing of ATF4 generally impairs cell growth, as previously reported (Harding et al., 2003), but does not specifically affect H9c2 cell growth and survival upon AMA treatment when compared to DMSO (Figure 5C). Similarly, siRNA mediated silencing of CHOP reduces its baseline protein and RNA expression and completely prevents its induction by AMA (Figure 5F; Supplementary Figure S11C). Interestingly, the knockdown of CHOP increased ATF4 protein levels in unstressed (DMSO treated) cells but had no effect on the induction of ATF4 by AMA (Figure 5F). Importantly, silencing of CHOP does not affect growth and survival of H9c2 cells upon AMA treatment (Figure 5E). In summary, despite its robust activation the ISR is not required for growth and survival of H9c2 cells upon mitochondrial complex III inhibition.

### Silencing of HSF1 and HSP60 does not impair growth of H9c2 cells upon antimycin A treatment

**FIGURE 6:**
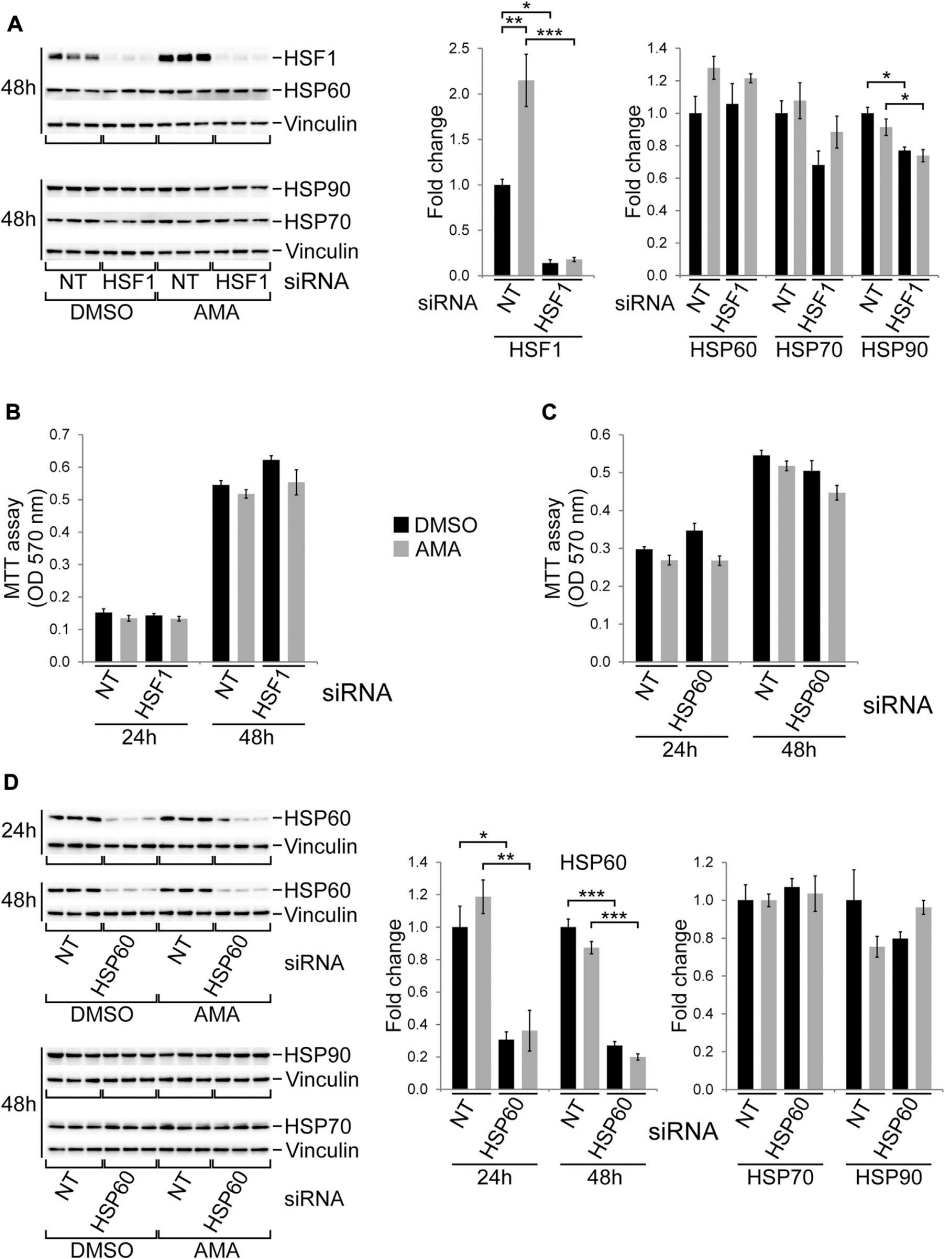
HSF1 and HSP60 are not required for growth and survival of H9c2 cells upon antimycin A treatment. **(A)** Western blots showing protein expression of HSF1 and various chaperones in H9c2 cells transfected with non-target (NT) or HSF1 siRNA and subsequently treated with DMSO or AMA (50 µM) for 24 and 48 h. **(B)** MTT assays of H9c2 cells treated as outlined in **(A)** [*n* = 3 wells per treatment in **(A)** and **(B)**]. **(C)** MTT assays of H9c2 cells transfected with non-target (NT) or HSP60 siRNA and subsequently treated with DMSO or AMA for 24 and 48 h. **(D)** Western blots showing protein expression of HSP60 and other chaperones in H9c2 cells treated as outlined in **(C)** [*n* = 3 wells per treatment in **(C)** and **(D)**]. (*p < 0.05, **p < 0.01, ***p < 0.001)

As described above HSF1, a key transcription factor mediating the HSR (Gomez-Pastor et al., 2018), is induced by AMA in H9c2 cells. To evaluate its role in cell growth and survival we silenced HSF1 by siRNA followed by DMSO or AMA treatment. Knockdown of HSF1 reduced its baseline protein levels and completely abolished the induction by AMA (Figure 6A), whereas it had no effect on the expression of HSP60 and HSP70 but mildly reduced protein levels of HSP90 (Figure 6A). Knockdown of HSF1 does not affect growth and survival of H9c2 cells upon AMA treatment when compared to cells transfected with non-target siRNA (Figure 6B).

The mitochondrial chaperone HSP60, although not altered by AMA in H9c2 cells (Supplementary Figure S6A), is markedly induced in embryonic cardiomyocytes upon complex III inhibition *in vivo* (Magarin et al., 2016). We therefore silenced HSP60 by siRNA in H9c2 cells, which efficiently reduces protein levels upon DMSO and AMA treatment without affecting HSP70 or HSP90 (Figure 6D). Knockdown of HSP60, however, had no major effect on growth and survival of AMA treated H9c2 cells (Figure 6C). In summary, silencing of HSF1 and HSP60 does not alter the susceptibility of H9c2 cells towards mitochondrial complex III inhibition.

### NRF2 is required for growth and survival of H9c2 cells upon antimycin A treatment

**FIGURE 7:**
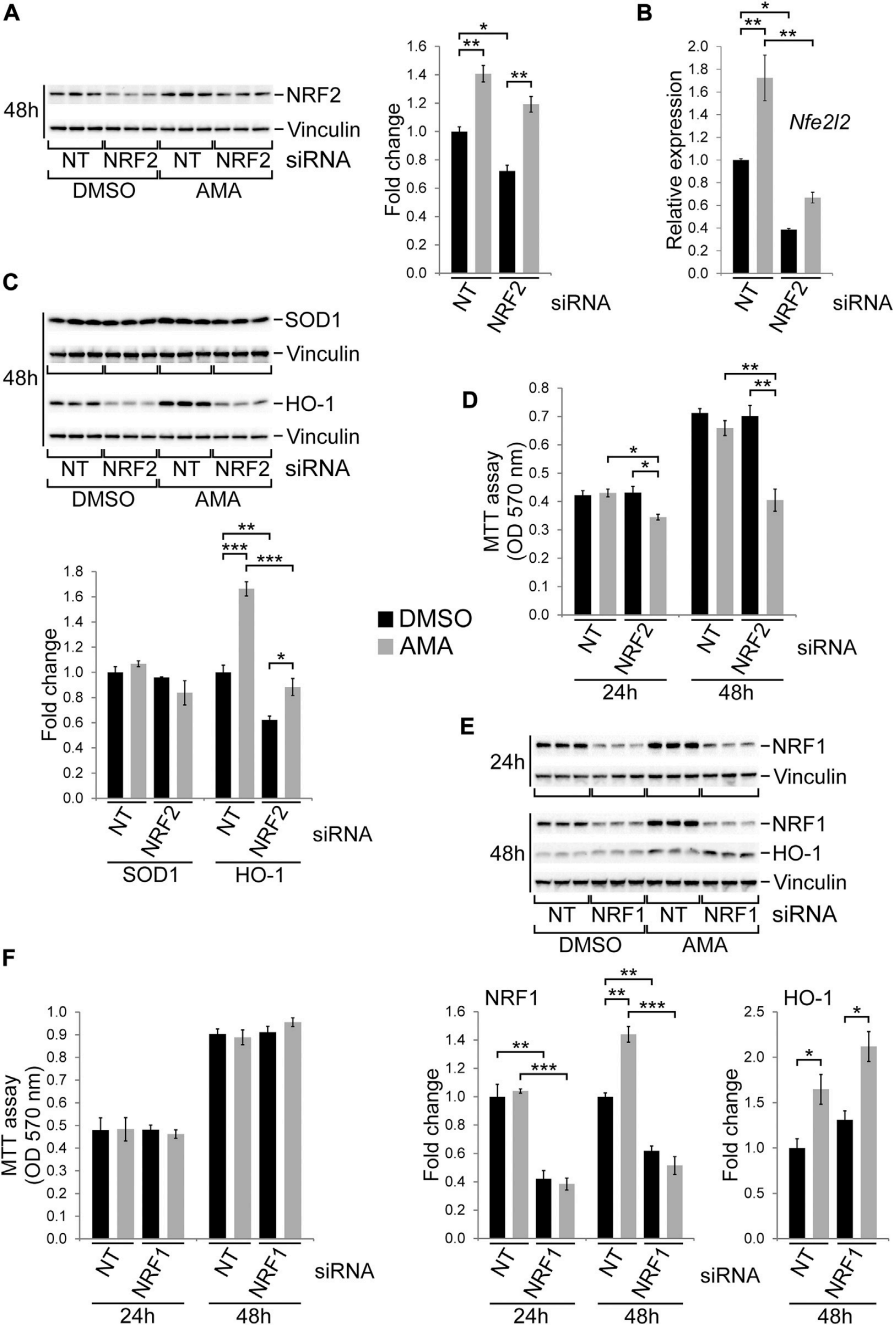
NRF2 is required for growth and survival of H9c2 cells upon antimycin A treatment. **(A)** Western blots showing protein expression of NRF2 in H9c2 cells transfected with non-target (NT) or NRF2 siRNA and subsequently treated with DMSO or AMA (50 µM) for 48 h. **(B)** qRT-PCR analyses showing RNA expression of Nfe2l2, the gene encoding NRF2, in H9c2 cells treated as outlined in **(A)**. **(C)** Western blots showing protein expression of the NRF2 target genes HO-1 and SOD1 in H9c2 cells treated as outlined in **(A)**. **(D)** MTT assays of H9c2 cells transfected with non-target (NT) or NRF2 siRNA and subsequently treated with DMSO or AMA for 24 and 48 h. **(E)** Western blots showing protein expression of NRF1 and HO-1 in H9c2 cells transfected with non-target (NT) or NRF1 siRNA and subsequently treated with DMSO or AMA for 24 h or 48 h. **(F)** MTT assays of H9c2 cells transfected with non-target (NT) or NRF1 siRNA and subsequently treated with DMSO or AMA for 24 and 48 h. [*p < 0.05, **p < 0.01, ***p < 0.001, *n* = 3 wells per treatment in **(A**–**E)**, *n* = 4 in **(F)**].

The transcription factor NRF2 is generally regarded as cell protective as it regulates a range of target genes involved in antioxidative defense such as HO-1 or SOD1 (Chen and Maltagliati, 2018). Given that NRF2 and some of its target genes are induced by AMA in H9c2 cells (Figure 3C) we silenced it by siRNA followed by AMA treatment. Knockdown efficiency for NRF2 was sufficient on the RNA level (Figure 7B) but moderate on the protein level (Figure 7A) which could be due to technical issues with the antibody used. Importantly, however, protein levels of HO-1 were clearly reduced by the NRF2 knockdown and its induction by AMA was completely prevented (Figure 7C). In contrast, SOD1 protein levels were unaffected by NRF2 siRNA transfection (Figure 7C). Silencing of NRF2 significantly reduced growth and survival of H9c2 cells upon AMA treatment (Figure 7D). The closely related family member NRF1 has similar cell protective functions and a partially overlapping target gene profile thereby also regulating cellular redox homeostasis (Biswas and Chan, 2010). We therefore silenced NRF1 by siRNA which results in efficient gene knockdown and completely prevents an induction of NRF1 protein expression by AMA after 48 h (Figure 7E). Interestingly, HO-1 protein levels were unaffected by the knockdown of NRF1 (Figure 7E) in contrast to NRF2 silencing (Figure 7C). Importantly, the knockdown of NRF1 does not alter growth and survival of AMA treated H9c2 cells over a period of 48 h (Figure 7F). In conclusion, NRF2 is involved in the tolerance of H9c2 cells towards oxidative stress caused by mitochondrial complex III inhibition.

### Small molecules activating stress response signaling failed to rescue HL-1 cells from antimycin A induced growth arrest

We aimed at rescuing or at least attenuating growth arrest and cell death in AMA treated HL-1 cells by pre-activation of cellular stress response signaling. Salubrinal activates the ISR (Boyce et al., 2005) and has been proposed to protect cardiomyocytes (Li et al., 2015). We confirmed that Salubrinal increases eIF2α phosphorylation, CHOP protein levels as well as RNA expression of *Ddit3*, *Asns* and *Trib3* in HL-1 cells (Supplementary Figures S12A,B). However, neither pre- nor co-incubation with Salubrinal protects HL-1 cells from growth arrest induced by AMA (Supplementary Figures S12C,D). In fact, long term Salubrinal treatment seems to have a growth inhibitory effect by itself and rather worsens growth and survival of HL-1 cells upon AMA co-treatment (Supplementary Figure S12D).

Celastrol has been shown to activate the HSR and upregulate heat shock proteins as well as HO-1 (Der Sarkissian et al., 2014). It has furthermore been proposed to be cardioprotective although this likely involves various cellular mechanisms (Sharma et al., 2015). Similarly, geranylgeranylacetone (GGA) induces small heat shock proteins (Marunouchi et al., 2014) and has been proposed to improve heart function after myocardial infarction (Shinohara et al., 2007). We found that Celastrol and GGA dose dependently impair growth and survival of HL-1 cells, likely because both induce oxidative stress themselves. However, we established a concentration that did not affect HL-1 cell growth (Supplementary Figures S13A,B) while still increasing protein levels of heat shock or antioxidative proteins (Supplementary Figure S13C). Notably, HO-1 was strongly induced by GGA whereas the effect of Celastrol was moderate (Supplementary Figure S13D). Nevertheless, neither pre- nor co-incubation with Celastrol or GGA were able to substantially alleviate growth arrest in HL-1 cells induced by AMA (Supplementary Figures S13E,F). Pre-incubation with Celastrol seems to be moderately protective after 24 h but this effect was not sustained after 48 h (Supplementary Figure S13E). In contrast, pre- and co-incubation with GGA rather worsen growth and survival of HL-1 cells upon AMA co-treatment for 24 h (Supplementary Figure S13F).

CDDO is a potent inducer of NRF2 signaling which has been shown to be protective in different organs including the heart by ameliorating oxidative stress response (Ichikawa et al., 2009; Tomlinson et al., 2019). Although CDDO itself dose dependently reduces HL-1 cell growth we identified a concentration that is tolerated (Supplementary Figure S14A) while robustly inducing the NRF2 targets HO-1 and SOD1 (Supplementary Figure S14B). However, neither pre- nor co-incubation with CDDO affects growth arrest in HL-1 cells induced by AMA (Supplementary Figures S14C,D).

In summary, preconditioning by mildly activating cellular stress response mechanisms does not protect HL-1 cells from subsequent mitochondrial complex III inhibition.

### Reactive oxygen species scavenging and metabolite supplementation failed to rescue HL-1 cells from antimycin A induced growth arrest

If oxidative stress was the main reason for growth arrest and death of AMA treated HL-1 cells then scavenging ROS by N-acetyl cysteine (NAC) might alleviate the latter. Co-treatment with various concentrations of NAC, however, did not have an effect on growth and survival of AMA treated HL-1 cells (Supplementary Figure S15). There appears to be a trend towards mild protection by low (1 mM) rather than high NAC concentrations but the respective difference between co-treatment and AMA alone does not reach statistical significance (Supplementary Figure S15).

It is well established that medium supplementation with pyruvate augments growth of certain cell types affected by mitochondrial dysfunction (King and Attardi, 1989). It has furthermore been proposed that this is due to pyruvate mediated stimulation of aspartate synthesis which is required to normalize proliferation of cells harboring mitochondrial defects (Birsoy et al., 2015). Nevertheless, neither pyruvate nor aspartate supplementation was able to reverse growth arrest and death of HL-1 cells subjected to complex III inhibition by AMA (Supplementary Figures S16A,B). High concentrations of aspartate by itself reduced HL-1 cell growth, likely by acidification of the culture medium.

## Discussion

This study shows that immature cardiomyoblasts largely tolerate mitochondrial complex III but not complex I inhibition, the latter inducing cell cycle arrest and cell death. Thus, the site of interrupted electron transport along the respiratory chain determines cell fate. It is well established that complex I and III are the main sites of superoxide production under both physiological as well as pathological conditions (Brand, 2010). Our data show similar ROS levels upon ROT and AMA treatment suggesting that the amount of superoxide production itself is not the main determinant of cell fate. It has been shown, however, that the distribution of superoxides within the cell differs depending on the site of production. Whereas complex I releases superoxides primarily into the mitochondrial matrix, complex III derived superoxides are distributed in the matrix as well as the intermembrane space (Muller et al., 2004). From the intermembrane space ROS can be released into the cytoplasm *via* voltage dependent anion channels (Han et al., 2003), which to a certain extent has also been described for complex I derived superoxides, however (Lustgarten et al., 2012). Nevertheless, it seems plausible that scavenging of complex III derived superoxides can be more effectively spread over various cellular compartments thereby benefiting from a higher overall antioxidative capacity (Dey et al., 2016). In contrast, scavenging of complex I derived superoxides mainly relies on antioxidative mechanisms in the mitochondrial matrix, whose capacity could quickly be overwhelmed leading to oxidative damage, mitochondrial membrane permeabilisation and cell death. Interestingly, it has been proposed that after ischemia and reperfusion in Langendorff perfused mouse hearts ROS generated from complex I have deleterious effects on functional recovery and infarct size but complex III derived ROS do not (Madungwe et al., 2016). Apart from handling oxidative stress it also has to be considered that metabolic reprogramming and anaplerosis (Des Rosiers et al., 2011) might be differentially affected in ROT versus AMA treated H9c2 cells thereby influencing cell fate. For instance, it has been shown that an enhanced glutamine metabolism is critical for survival of neonatal rat cardiomyocytes treated with H_2_O_2_ (Watanabe et al., 2021). Whether such metabolic adaptations differ for ROS endogenously produced from mitochondrial complex I versus III is currently unknown.

The molecular stress response signature in H9c2 cells following complex I versus complex III inhibition is remarkably similar and does not necessarily represent the rather drastic differences in cell growth and survival. The most striking finding was a much higher induction of CHOP in ROT compared to AMA treated H9c2 cells (Figure 3E). Interestingly, this was furthermore observed in OLI compared to AMA treated cells (Supplementary Figure S8B). Considering that OLI does impair H9c2 growth and shows an intermediate effect between AMA and ROT, it is tempting to speculate that CHOP might determine cell fate in response to various mitochondrial inhibitors. This is furthermore supported by one AMA lot which was found to impair H9c2 cell growth, as it also dramatically increases CHOP and ATF4 levels compared to AMA without growth inhibitory effects (Supplementary Figure S8A). Considering that CHOP can inhibit cell cycle progression and induce apoptosis upon activation of the UPR/ISR (Pakos-Zebrucka et al., 2016; Shpilka and Haynes, 2018), it might be an important determinant of cell fate in response to mitochondrial dysfunction. On the other hand, CHOP has been shown to be involved in the adaptive phase of the UPR^mt^ by regulating expression of a variety of genes required to restore mitochondrial protein homeostasis (Zhao et al., 2002; Aldridge et al., 2007). In addition, a recent study in the heart proposed that CHOP is required for adaptation to mitochondrial dysfunction by tuning the ISR and preventing excessive activation of the ATF4 transcriptional program (Kaspar et al., 2021). Our results show that CHOP knockdown increases ATF4 protein levels in unstressed H9c2 cells (Figure 5F) but does not alter their growth and survival upon AMA treatment (Figure 5E). Thus, whether excessive CHOP expression in ROT and OLI treated H9c2 cells contributes to cell death and growth arrest or represents an attempt to regulate and adapt the mitochondrial stress response is currently unclear.

Our data suggest that the UPR/ISR as well as the HSR are not primarily responsible for the tolerance of H9c2 cells towards mitochondrial complex III inhibition. In contrast, knockdown of NRF2 decreases growth and survival of AMA treated H9c2 cells thereby effectively increasing its cytotoxicity. NRF2 is a key transcription factor regulating the expression of multiple genes involved in redox homeostasis and antioxidative defense in various organs including the heart (Chen and Maltagliati, 2018). It has been shown to be cardioprotective in a variety of *in vitro* and *in vivo* settings (Zhu et al., 2008; He et al., 2009; Li et al., 2009). Consequently, activation of NRF2 signaling has been an attractive therapeutic target leading to clinical trials for different diseases (Robledinos-Antón et al., 2019) while at the same time fostering preclinical studies exploring the effects on cardiovascular disease. Indeed, small molecules that increase NRF2 protein levels have been shown to protect cardiomyocytes from oxidative stress *in vitro* and *in vivo* (Ichikawa et al., 2009; Xing et al., 2012; Tomlinson et al., 2019). The latter also includes protective functions within H9c2 cells after hypoxia and reoxygenation (Li et al., 2017) or in response to doxorubicin induced toxicity (Ye et al., 2019). Given that the majority of insults applied in these studies directly or indirectly increase oxidative stress in cardiac mitochondria, our data confirm that mitochondrial dysfunction by complex III inhibition requires NRF2 for normal growth and survival of H9c2 cells. Importantly, the closely related transcription factor NRF1 protects H9c2 cells from hypoxia induced cell death (Li et al., 2021) and has recently been shown to be a major driver of myocardial regeneration in mice by regulating proteostasis and redox balance (Cui et al., 2021). Our data, however, revealed no detectable effect of NRF1 silencing on growth and survival of AMA treated H9c2 cells, suggesting that NRF2 is primarily responsible for redox homeostasis of immature cardiomyocytes upon mitochondrial complex III inhibition. Among the known NRF2 target genes with antioxidative potential HO-1 stood out in this study given that it showed equally high expression in immature H9c2 compared to differentiated HL-1 cells, was robustly induced by AMA (as well as by ROT and OLI) and efficiently decreased following NRF2 knockdown. Importantly, HO-1 by itself has been shown to be cardioprotective (Otterbein et al., 2016) and involved in antioxidative defense and mitochondrial quality control in the heart (Yet et al., 2001; Hull et al., 2016). Whether HO-1 is specifically required for the cell protective effects in AMA treated H9c2 cells downstream of NRF2 will have to be addressed in future studies.

A key observation of this study is the striking difference in proliferation and survival of immature compared to differentiated cardiomyocyte cell lines subjected to mitochondrial complex III inhibition. Whereas H9c2 cells largely tolerate AMA treatment (up to 50 µM), HL-1 cells rapidly undergo cell cycle arrest and death at much lower concentrations (10 µM). Interestingly, H9c2 cells have been proposed to be more susceptible towards hypoxia/reoxygenation than HL-1 cells (Kuznetsov et al., 2015), suggesting that cell survival and stress response in both cell types might differ depending on the type of the insult. We have previously shown *in vivo* that embryonic cardiomyocytes in the mouse heart survive complex III inhibition imposed by the loss of cytochrome c (Drenckhahn et al., 2008; Magarin et al., 2016). Whereas these *in vivo* studies showed reduced proliferation but no signs of cell death in embryonic cardiomyocytes, AMA treated H9c2 cells proliferate normally but moderately undergo caspase independent cell death. These differences are likely due to the *in vivo* versus *in vitro* setting and differences between H9c2 cells and true embryonic cardiomyocytes. It raises the possibility, however, that low levels of cell death where missed in cytochrome c deficient embryonic cardiomyocytes in our previous study. Nevertheless, cytochrome c deficient cardiomyocytes were detected in the neonatal and adult heart (Drenckhahn et al., 2008; Magarin et al., 2016), indicating that the majority survive the period of postnatal maturation and terminal differentiation.

The latter raises the question whether immature cardiomyocyte can adapt to unfavorable conditions by activating a highly versatile cellular stress program which can be maintained upon further differentiation. That way, embryonic and fetal cardiomyocytes not only withstand mitochondrial dysfunction during heart development but survive and remain functional until adult stages. This would represent an intrauterine preconditioning or programming of stress resistance which equips the cell to better cope with unfavorable conditions after terminal differentiation. We have previously shown, for instance, that the mitochondrial chaperone HSP60 is induced in embryonic cardiomyocytes upon cytochrome c deficiency and this high expression is maintained in these cells in the neonatal and adult heart (Magarin et al., 2016). This furthermore raises the question when exactly during cardiomyocyte differentiation the plasticity in orchestrating various cellular stress response mechanisms is lost. Whether this coincides with the loss of cell cycle activity or the switch from glycolysis to an oxidative metabolism after birth (Bongiovanni et al., 2021; Garbern and Lee, 2021) will have to be evaluated in future studies, for example using *in vitro* differentiation of iPS derived cardiomyocytes. H9c2 cells can be differentiated towards a more cardiomyocyte-like phenotype under defined culture conditions. Interestingly, such differentiation increases their susceptibility towards doxorubicin and isoproterenol induced cytotoxicity (Branco et al., 2011; Branco et al., 2012). The latter appears to be associated with differences in mitochondrial integrity as well as activation of stress response and survival pathways in differentiated versus immature H9c2 cells (Branco et al., 2013). Importantly, mitochondrial biogenesis, turnover, structure and function change in the prenatal versus adult heart which also alters ROS detoxification capacity and protein quality control (Sánchez-Díaz et al., 2020; Garbern and Lee, 2021) thereby affecting cardiac function upon pathological conditions. If stress tolerance is lost upon terminal differentiation of cardiomyocytes this raises the interesting hypothesis that reactivation of an embryonic stress response program would protect adult cardiomyocytes from pathological conditions. We show here, however, that NRF2 is essential for growth and survival of H9c2 cells upon AMA treatment whereas activation of NRF2 signaling by the small molecule CDDO does not protect HL-1 cells. This suggests that activating a single cell protective gene or signaling pathways is insufficient to rescue differentiated cardiomyocytes from mitochondrial dysfunction. The latter likely requires the orchestration of different stress response, cell survival and antioxidative defense mechanisms, thereby highlighting the complexity of therapeutic approaches aiming at cardioprotection in the adult heart.

A high stress tolerance of immature cardiomyocytes as shown for mitochondrial complex III inhibition *in vivo* (Magarin et al., 2016) and *in vitro* (this study) might be essential for growth plasticity of the heart during intrauterine development. The embryo and fetus can be exposed to various insults during pregnancy resulting in either transient or even persisting unfavorable growth conditions and intrauterine growth restriction (IUGR). The latter include maternal mal- or undernutrition, maternal disease (including infections, obesity, diabetes, hypertension, anemia, cardiovascular disease, etc.) or drug and alcohol abuse during pregnancy as well as placental insufficiency or genetic factors affecting the fetus itself. The majority of these insults effectively impair nutrient and oxygen supply to the embryo and fetus or result in the exposure to harmful and cytotoxic substances. By possessing a highly versatile stress response program embryonic and fetal cardiomyocytes might be able to compensate the resulting unfavorable cellular conditions thereby allowing progression of cardiac development and growth. Any insult that impairs stress tolerance in immature cardiomyocytes during intrauterine development might therefore increase cell death rates or lead to cell cycle inhibition. This in turn, would increase the risk of structural cardiac defects (i.e. congenital heart disease) or result in an incomplete cardiomyocyte complement at birth. The latter has been proposed to be an important determinant of cardiovascular health during the life course (Botting et al., 2012). A precise understanding of molecular mechanisms regulating stress response and survival in embryonic and fetal cardiomyocytes might therefore indicate new approaches for the prevention of cardiovascular disease originating during intrauterine development.

A limitation of this study is the use of cardiac cell lines which exhibit various differences in function and biology compared to primary cardiomyocytes. H9c2 cells, although originally derived from the embryonic rat heart and widely used as *in vitro* model for cardiomyocytes, do not assemble sarcomeres and are non-contractile (Kimes and Brandt, 1976; Hescheler et al., 1991) such that they do not experience the same metabolic and mechanical demands compared to embryonic cardiomyocytes. The latter could cause differences in stress tolerance and cell survival. HL-1 cells are contractile cells from a murine atrial tumor which have been genetically modified to allow rapid proliferation (Claycomb et al., 1998). This does not necessarily represent terminally differentiated ventricular cardiomyocytes that had undergone cell cycle arrest. Whether constant cell division, for example, alters the susceptibility of HL-1 cells towards mitochondrial dysfunction compared to adult cardiomyocytes is unknown. We therefore cannot exclude that activation of cell protective mechanisms that failed to recue HL-1 cells from mitochondrial complex III inhibition might be more successful in primary adult cardiomyocytes. Furthermore, a large number of small molecules and compounds not included in this study are well suited candidates to protect HL-1 cells upon AMA treatment. For example, the ROS scavenger MitoTEMPO, which is specifically targeted to mitochondria, exhibits protection in cardiomyocytes subjected to oxidative stress (Dey et al., 2018; Peoples et al., 2019) and might therefore be more effective than NAC used in this study. Consequently, future *in vitro* experiments potentially involving high-throughput screening of small molecule libraries using primary embryonic and adult cardiomyocytes or iPSC derived cardiomyocytes as well as *in vivo* approaches will have to confirm the findings of this study.

Another limitation is the observation that one particular AMA lot used throughout the course of this study inhibited growth of H9c2 cells whereas three other lots from different suppliers did not. Although this one lot seemed to induce much higher stress levels likely leading to cell cycle arrest (but not excessive cell death) we did not identify the underlying molecular mechanism. AMA is not a synthetic and highly purified compound but is isolated from bacteria of the *Streptomyces* family. Furthermore, it is a mixture of up to 40 known isoforms whose exact composition differs in each AMA lot. We therefore speculate that *gi*AMA might contain isoforms with altered potency towards complex III inhibition or with additional off-target effects. Alternatively, contamination with other bacterial components during the isolation process could have an effect on H9c2 cell proliferation.

In summary, our data identified a striking stress tolerance of immature cardiomyoblasts towards mitochondrial complex III inhibition, which at least in part relies on redox homeostasis regulated by NRF2. In differentiated cardiomyocytes complex III inhibition results in cell cycle arrest and death which cannot be rescued by various small molecules that activate cell protective mechanisms or attenuate oxidative stress. These findings might have important implications for embryonic and fetal cardiac growth plasticity in response to fluctuating intrauterine conditions to allow formation of a fully functional heart and prevent congenital heart disease. On the other hand, our data highlight the complexity of cardioprotective therapeutic approaches in the adult heart whose translation into the clinic has been challenging despite a multitude of promising preclinical studies.

## Data Availability

The original contributions presented in the study are included in the article/Supplementary Material, further inquiries can be directed to the corresponding author.
