# Supplementary material for: Inhibition of mitochondrial respiration has fundamentally different effects on proliferation, cell survival and stress response in immature versus differentiated cardiomyocyte cell lines
Source: Front Cell Dev Biol. 2022 Sep 23;10:1011639. doi: 10.3389/fcell.2022.1011639 (PMC9538794; doi:10.3389/fcell.2022.1011639)
Supplement: Supplementary file 1 [file DataSheet1.PDF]

Supplementary Material

Supplementary Figures

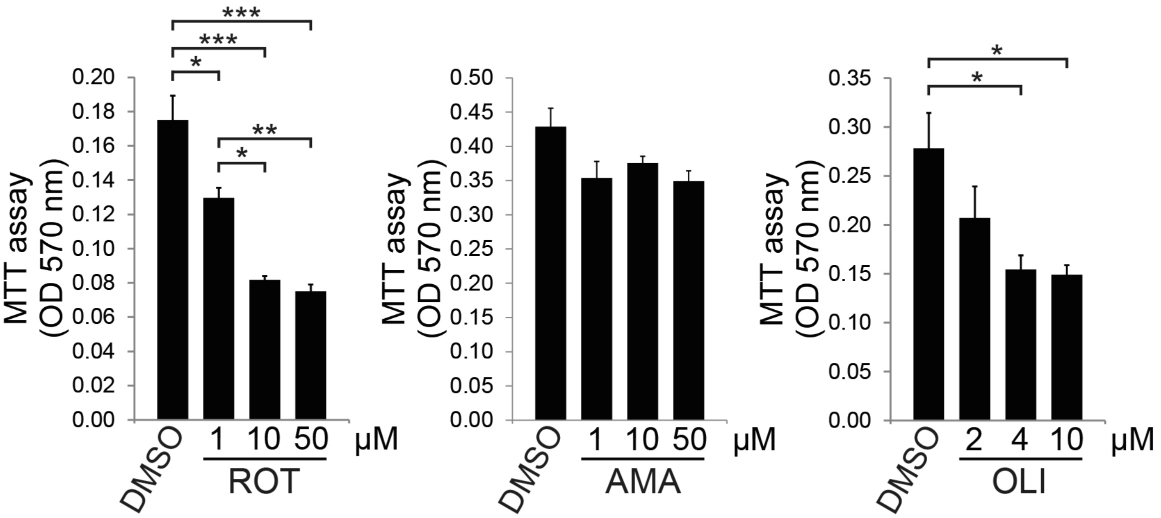

Supplementary Figure 1. Growth and survival of H9c2 cells treated with different inhibitors of mitochondrial respiration. MTT assays of H9c2 cells treated with DMSO as control and the indicated concentrations of the mitochondrial complex I inhibitor rotenone (ROT), the complex III inhibitor antimycin A (AMA) and the complex V inhibitor oligomycin (OLI) for 24h. (**P*<0.05, ***P*<0.01, ****P*<0.001, n=3 wells per treatment)

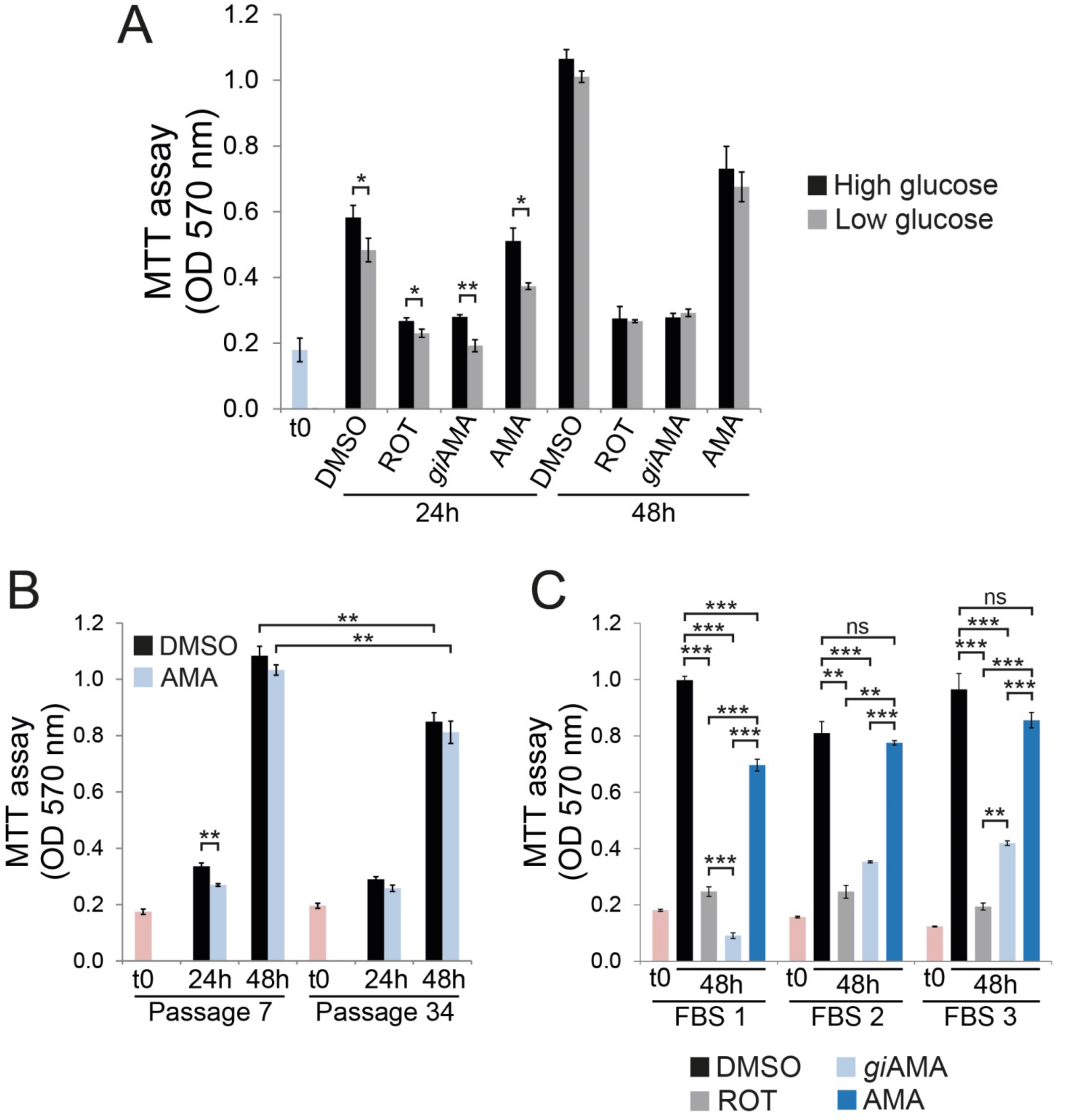

Supplementary Figure 2. Evaluation of culture conditions to inhibit mitochondrial respiration in H9c2 cells. (A) MTT assays of H9c2 cells cultured in medium containing high (4500 mg/L) and low (1000 mg/L) glucose concentrations and treated with DMSO, ROT (10 µM) or two different lots of AMA (50 µM) for 24h and 48h. (B) MTT assays of H9c2 cells from passage 7 or 34 treated with DMSO and AMA (50 µM), respectively, for 24h and 48h. (C) MTT assays of H9c2 cells cultured in three different batches of fetal bovine serum (FBS1 to 3) and treated with DMSO, ROT (10 µM) or two different lots of AMA (50 µM) for 48h. *gi*AMA in (A) and (C) is the same as lot A in Figure 1 showing the described growth inhibitory effects. (**P*<0.05, ***P*<0.01, ****P*<0.001, ns=non-significant, n=3 wells per treatment, t0 represents MTT values at the onset of treatment)

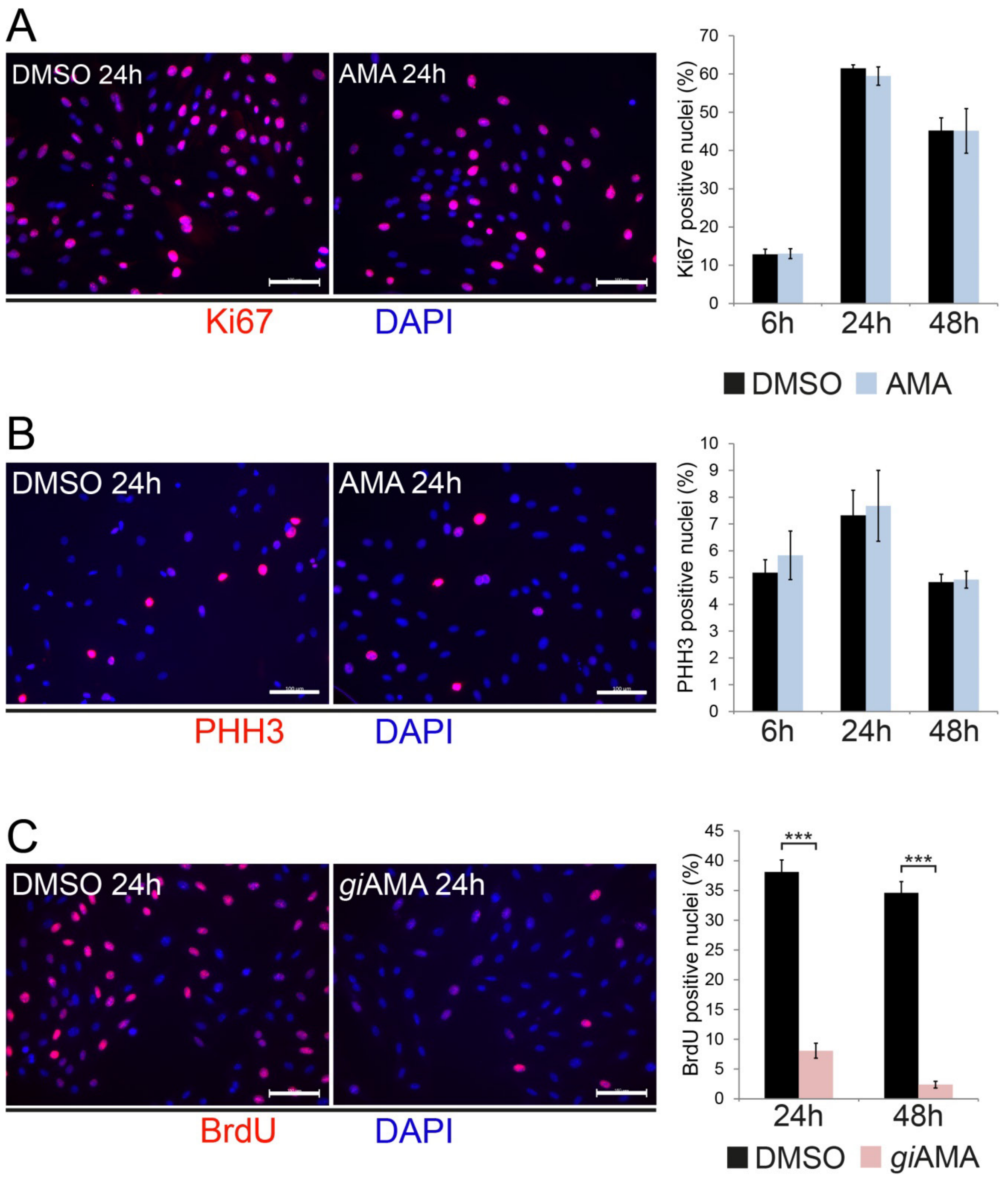

Supplementary Figure 3. Detection of cell cycle activity in H9c2 cells treated with rotenone and antimycin A. (A) Fluorescence microscopy images detecting the cell cycle marker Ki67 and (B) the mitosis marker phospho-histone H3 (PHH3) in H9c2 cells treated with DMSO or AMA (50 µM) for 6h, 24h and 48h. Cell cycle activity was evaluated as the number of Ki67 and PHH3 positive nuclei (both stained in red), respectively, related to the overall number of nuclei (stained in blue using DAPI, n=4 wells per treatment in (A) and (B)). (C) Fluorescence microscopy images showing BrdU incorporation (stained in red) in H9c2 cells treated with DMSO and 50 µM *gi*AMA (AMA lot A causing growth inhibitory effects as shown in Figure 1). Cell cycle activity was evaluated as described above for Ki67 and PHH3 (24h n=6 and 48h n=4 wells per treatment, ***P<0.001). Scale bar = 100 µm in all panels.

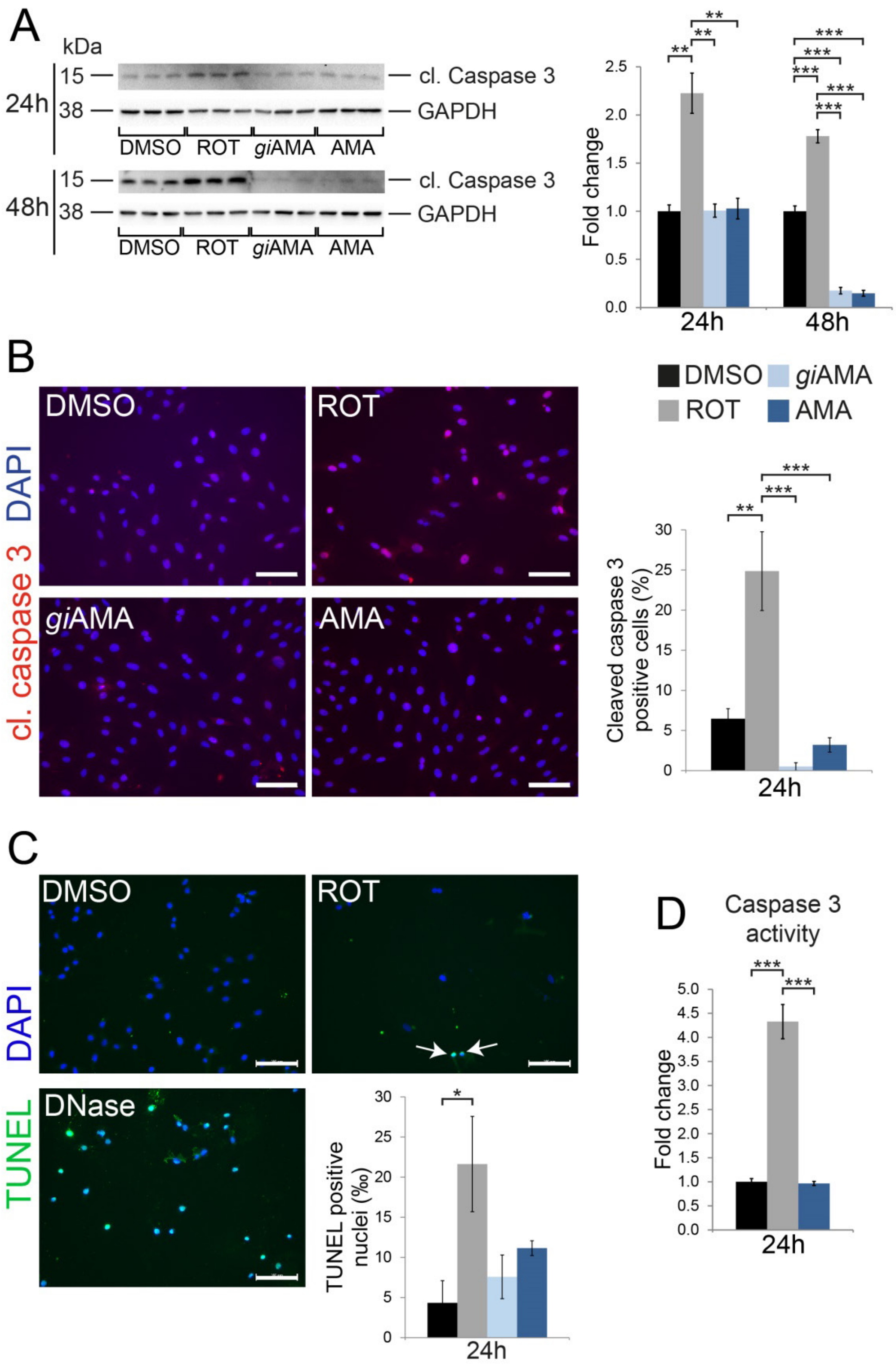

Supplementary Figure 4. Detection of apoptosis in H9c2 cells treated with rotenone and antimycin A. (A) Western blots for cleaved caspase 3 in H9c2 cells treated with ROT (10 µM) and two different lots of AMA (50 µM) for 24h and 48h (n=3 wells per treatment). (B) Immunofluorescence staining for cleaved caspase 3 (stained in red) in H9c2 cells treated with ROT and two different lots of AMA for 24h. (C) Representative fluorescence microscopy images showing

TUNEL staining in H9c2 cells treated with DMSO or ROT for 24h. DNase treatment of cells prior to TUNEL staining was included as positive control for experimental procedures of the assay. TUNEL images for AMA are not shown, as they are undistinguishable from DMSO. Apoptosis rates were evaluated as the number of TUNEL positive nuclei (stained in green, see arrows in (C), n=3 wells per treatment) or cleaved caspase 3 positive cells (n=4 wells per treatment) related to the overall number of nuclei (stained in blue using DAPI). (D) H9c2 cells were treated with DMSO, ROT or AMA for 24h (n=3 wells per treatment) and cell lysates were incubated with the fluorogenic caspase 3 substrate Ac-DEVD-AMC. Fluorescence intensity was determined in a plate reader, normalized to protein content and the resulting caspase 3 activity was related to DMSO treated control cells. (*$P<0.05$, **$P<0.01$, ***$P<0.001$, scale bar = 100 µm in (B) and (C); *gi*AMA in (A) to (C) is the same as lot A in Figure 1 showing the described growth inhibitory effects.)

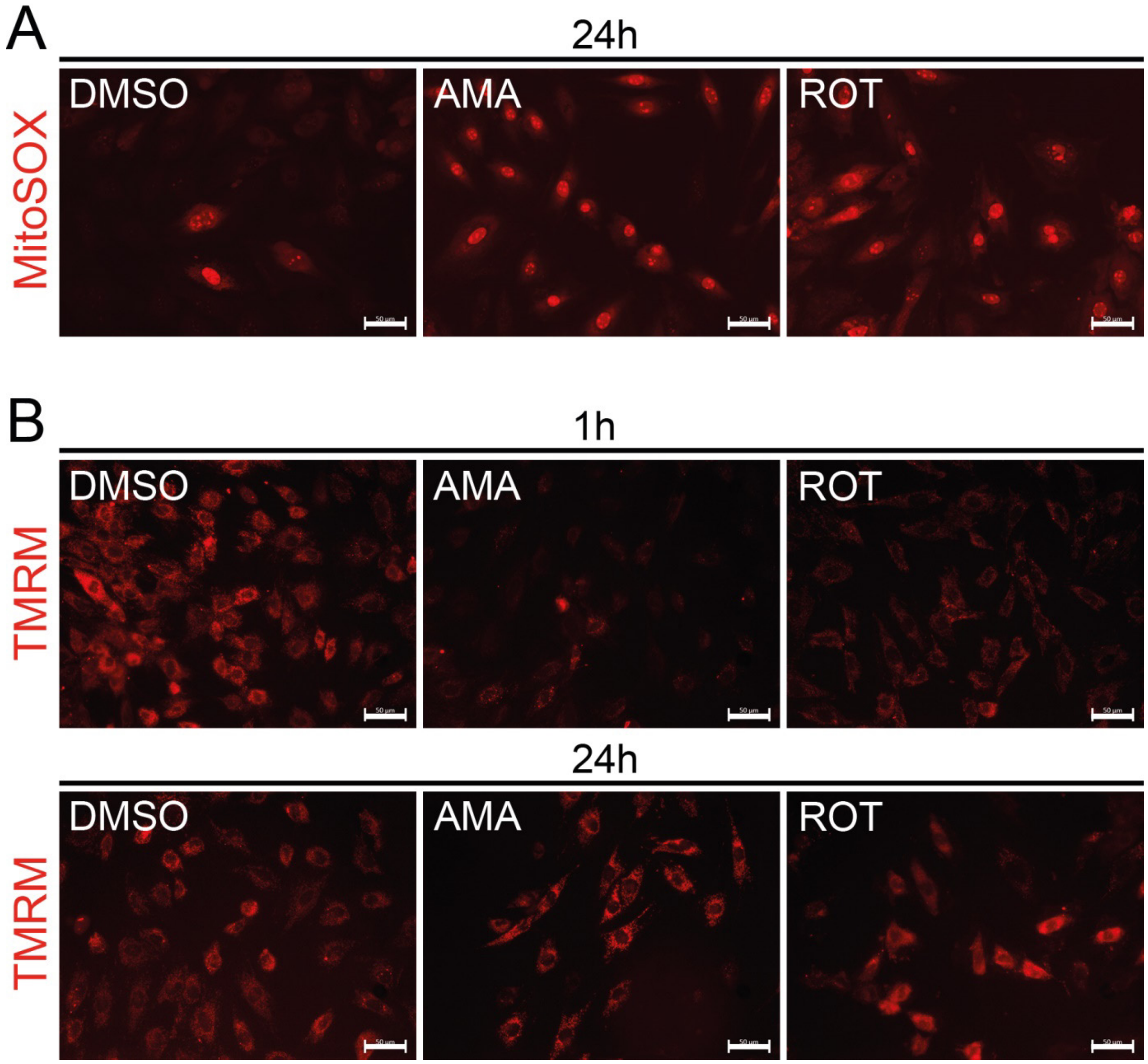

Supplementary Figure 5. Mitochondrial ROS generation and membrane potential in H9c2 cells treated with rotenone and antimycin A. (A) Fluorescence microscopy images of H9c2 cells treated with DMSO, ROT (10 µM) and AMA (50 µM) for 24h and subsequently incubated with MitoSOX, a red fluorescent probe for reactive oxygen species generated inside mitochondria. MitoSOX positive cells are much more abundant upon ROT and AMA treatment compared to DMSO, even though nuclear staining indicates unintended leakage of the oxidized probe from mitochondria followed by binding to nuclear DNA (scale bar = 50 µm). (B) Fluorescence microscopy images of H9c2 cells treated with DMSO, ROT and AMA for 1h and 24h and subsequently incubated with TMRM (Tetramethylrhodamin-methylester), a fluorescent sensor for mitochondrial membrane potential. TMRM accumulates in intact mitochondria resulting in red fluorescence but is lost upon membrane depolarization. Note reduced TMRM staining in H9c2 cells treated with AMA and ROT for 1h compared to DMSO, which is no longer evident after 24h (scale bar = 50 µm).

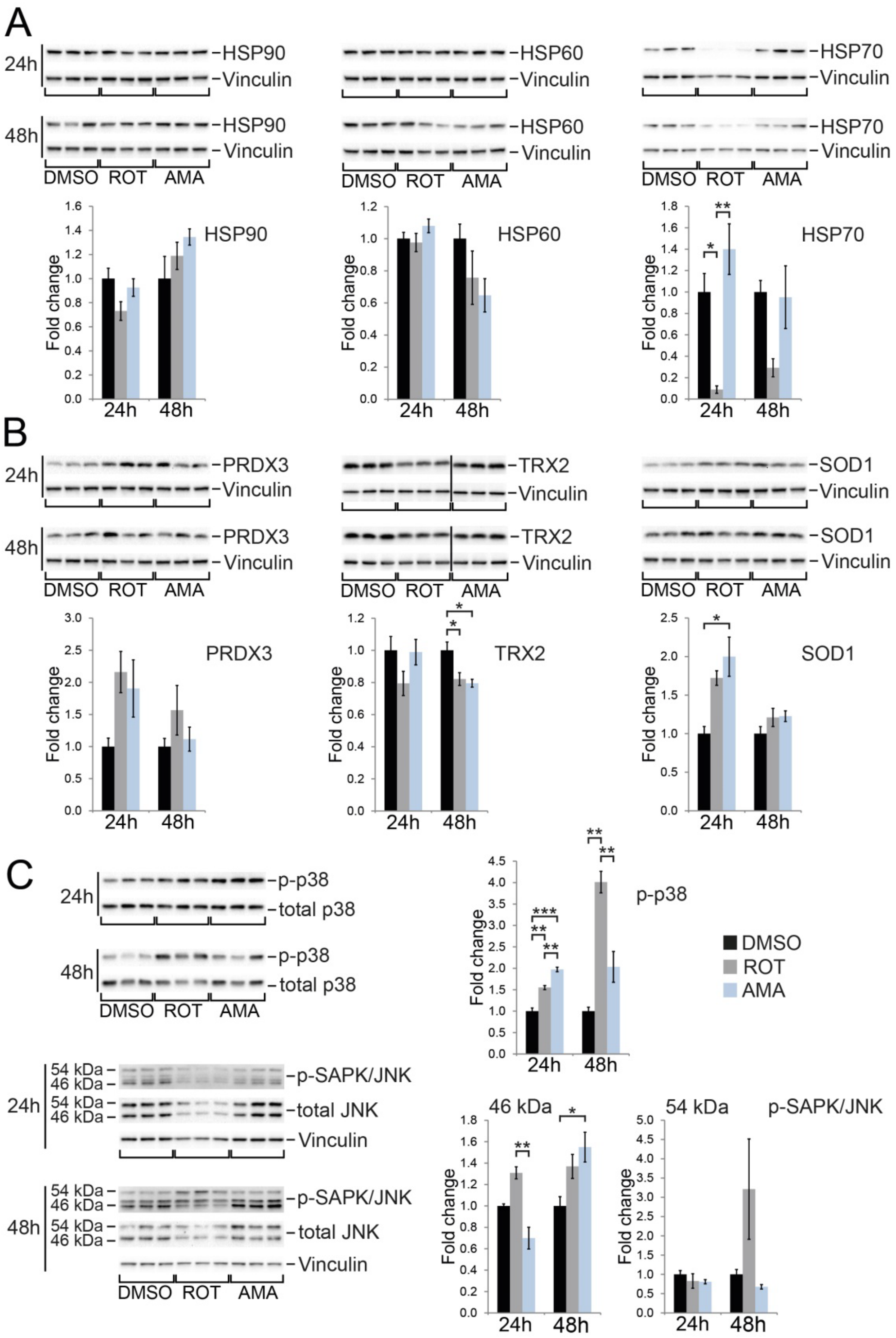

Supplementary Figure 6. Evaluation of stress response mechanisms in H9c2 cells treated with ROT (10 µM) or AMA (50 µM) for 24h and 48h. (A) Western blots showing protein expression of the chaperones HSP60, HSP70 and HSP90. (B) Western blots showing expression of antioxidative proteins PRDX3, TRX2 and SOD1 (a vertical black line indicates that samples were run on the same gel but were non-contiguous). (C) Western blots showing phosphorylation of the stress responsive MAP kinases p38 (Thr180/Tyr182) and SAPK/JNK (Thr183/Tyr185). (**P*<0.05, ***P*<0.01, ****P*<0.001, n=3 wells per treatment in (A) – (C))

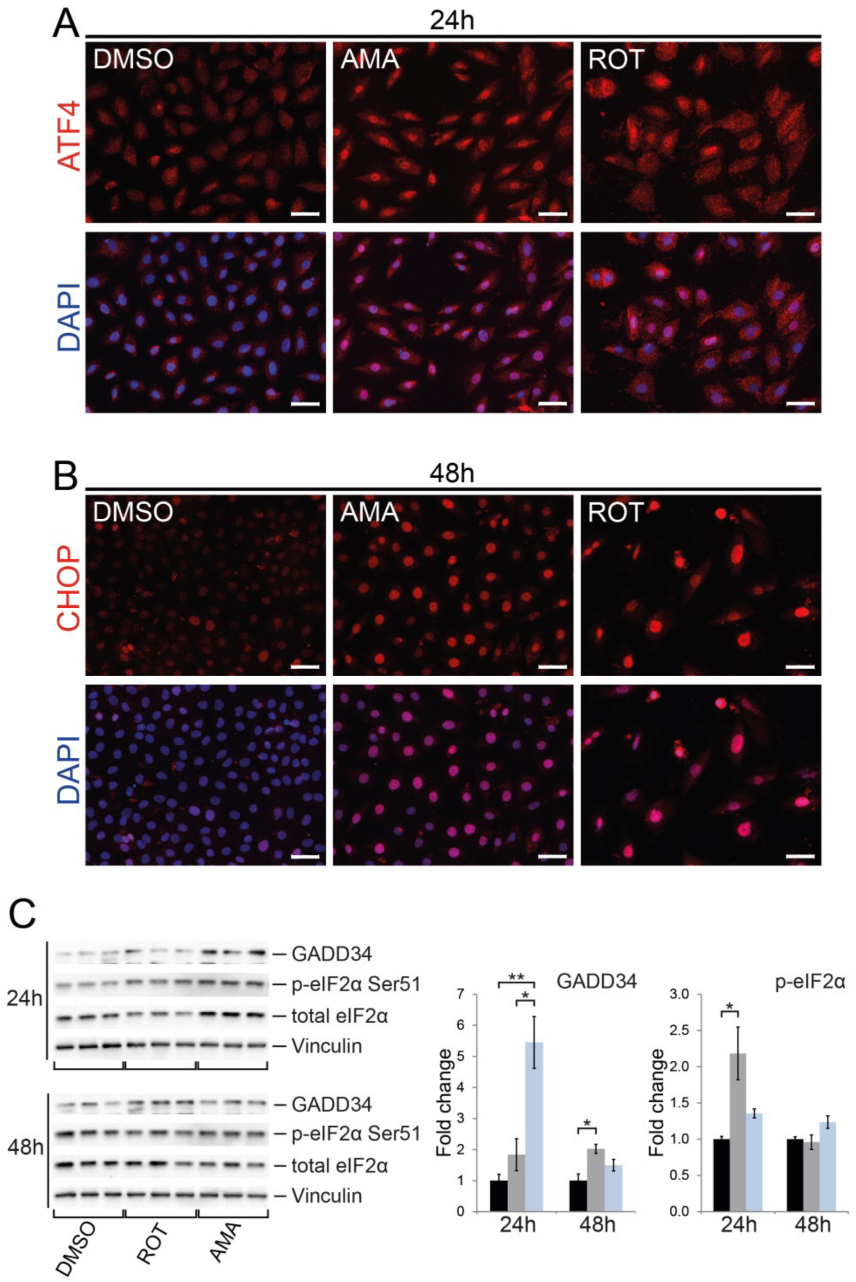

Supplementary Figure 7. Evaluation of the ISR in H9c2 cells treated with rotenone and antimycin A. (A) Representative fluorescence microscopy images of H9c2 cells treated with DMSO, ROT (10 µM) and AMA (50 µM) for 24h and subsequently stained with an antibody against ATF4 (red). (B) Representative fluorescence microscopy images of H9c2 cells treated with DMSO, ROT and AMA for 48h and subsequently stained with an antibody against CHOP (red). Note the prominent nuclear staining of both ATF4 and CHOP in AMA and ROT treated cells compared to DMSO. Nuclei are stained in blue using DAPI and scale bar = 50 µm in (A) and (B). (C) Western blots showing phosphorylation of eukaryotic initiation factor 2α (eIF2α) and the respective phosphatase GADD34 in H9c2 cells treated with ROT and AMA for 24h and 48h (*P<0.05, **P<0.01, n=3 wells per treatment).

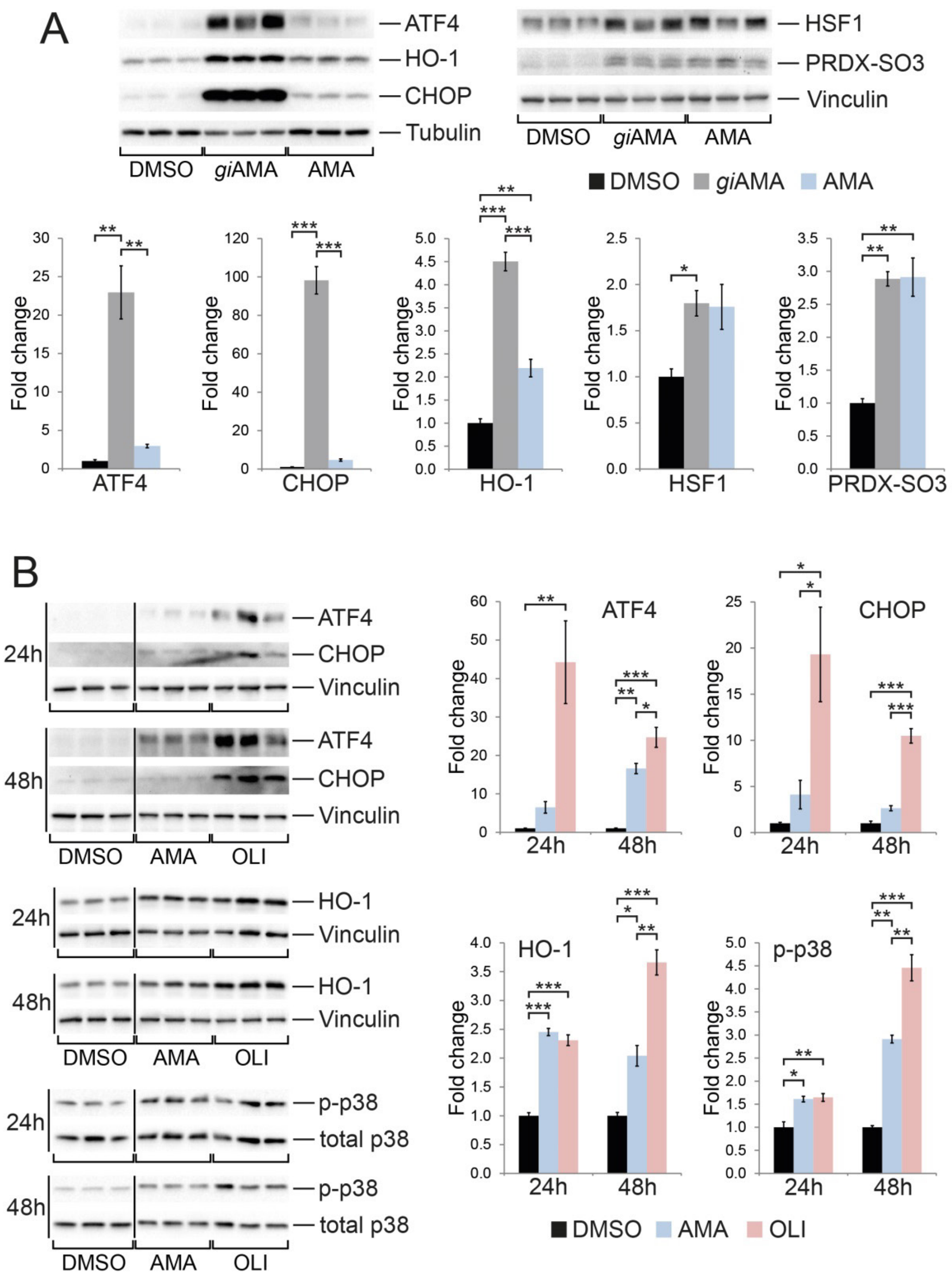

Supplementary Figure 8. Stress response in H9c2 cells treated with growth inhibitory antimycin A and oligomycin. (A) Western blots showing protein expression of the ISR transcription factors ATF4 and CHOP, the antioxidative enzyme HO-1, the HSR transcription factor HSF1 as well as hyperoxidized peroxiredoxin (PRDX-SO3) in H9c2 cells treated with two different lots of AMA (50 µM) for 24h. *gi*AMA corresponds to lot A in Figure 1, whereas AMA corresponds to lot B. Note the excessive induction of ATF4 and CHOP expression and the mildly increased HO-1 expression in *gi*AMA compared to DMSO and AMA treated cells, whereas HSF1 expression and PRDX oxidation is not different between AMA lots. (B) Western blots showing protein expression of the ISR

transcription factors ATF4 and CHOP, the antioxidative enzyme HO-1 as well as phosphorylation of stress responsive p38 MAP kinase in H9c2 cells treated with AMA (50 µM) or OLI (5 µM) for 24h and 48h. Note the marked induction of ATF4 and CHOP expression in OLI compared to DMSO and AMA treated cells at both time points. HO-1 expression and p38 phosphorylation are increased by OLI compared to DMSO and AMA after 48h but not 24h. (*$P<0.05$, **$P<0.01$, ***$P<0.001$, n=3 wells per treatment in (A) and (B))

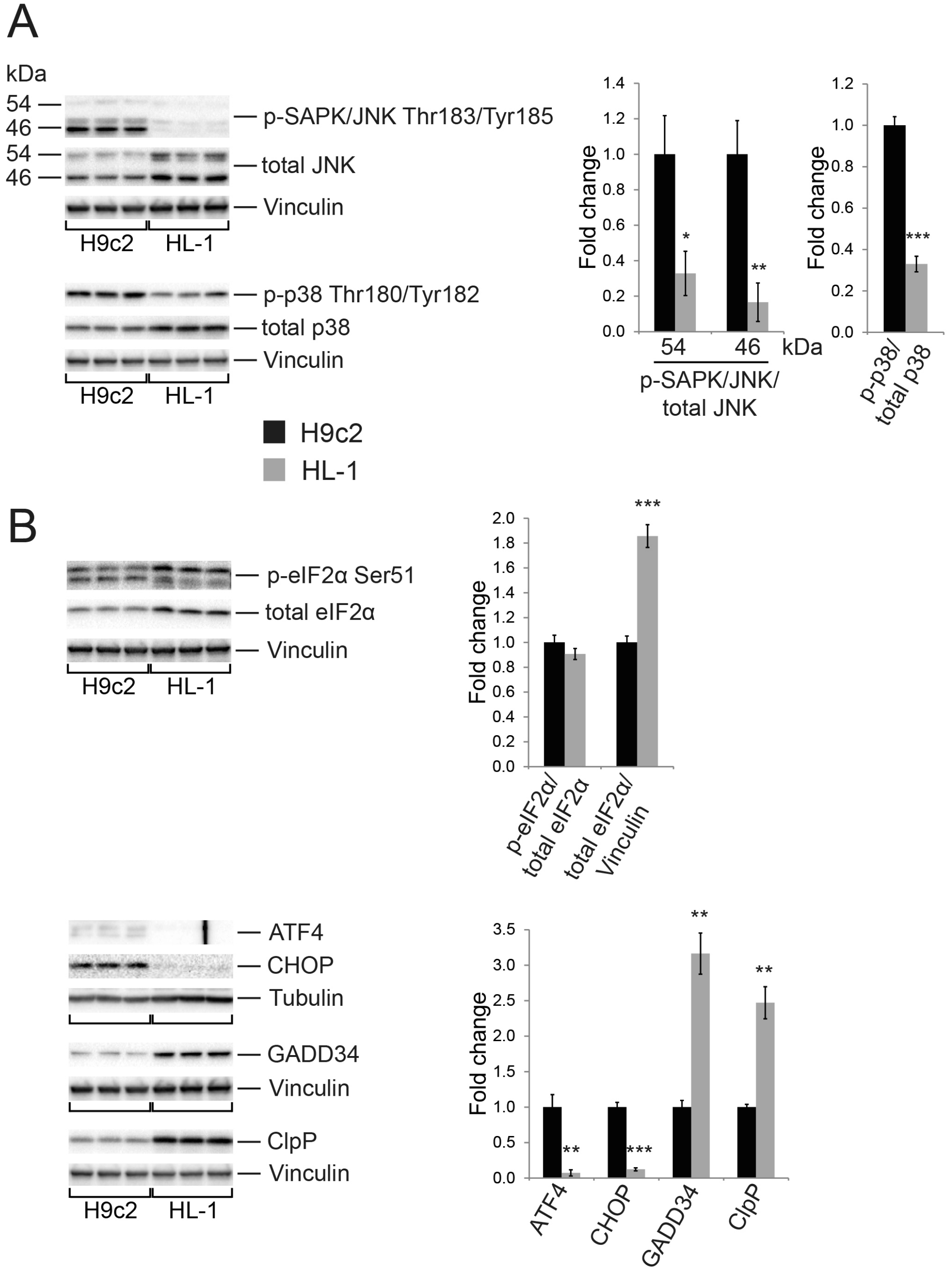

Supplementary Figure 9. Evaluation of stress response pathways in H9c2 versus HL-1 cells under baseline conditions. (A) Western blots showing phosphorylation of the stress responsive MAP kinases p38 and SAPK/JNK in H9c2 compared to HL-1 cells under baseline conditions. (B) Western blots showing components of the ISR (phosphorylation of eIF2α as well as expression of ATF4, CHOP and GADD34) and the UPR^{mt} (ClpP) in H9c2 compared to HL-1 cells under baseline conditions. (*P<0.05, **P<0.01, ***P<0.001, n=6 wells per group in (A) and (B))

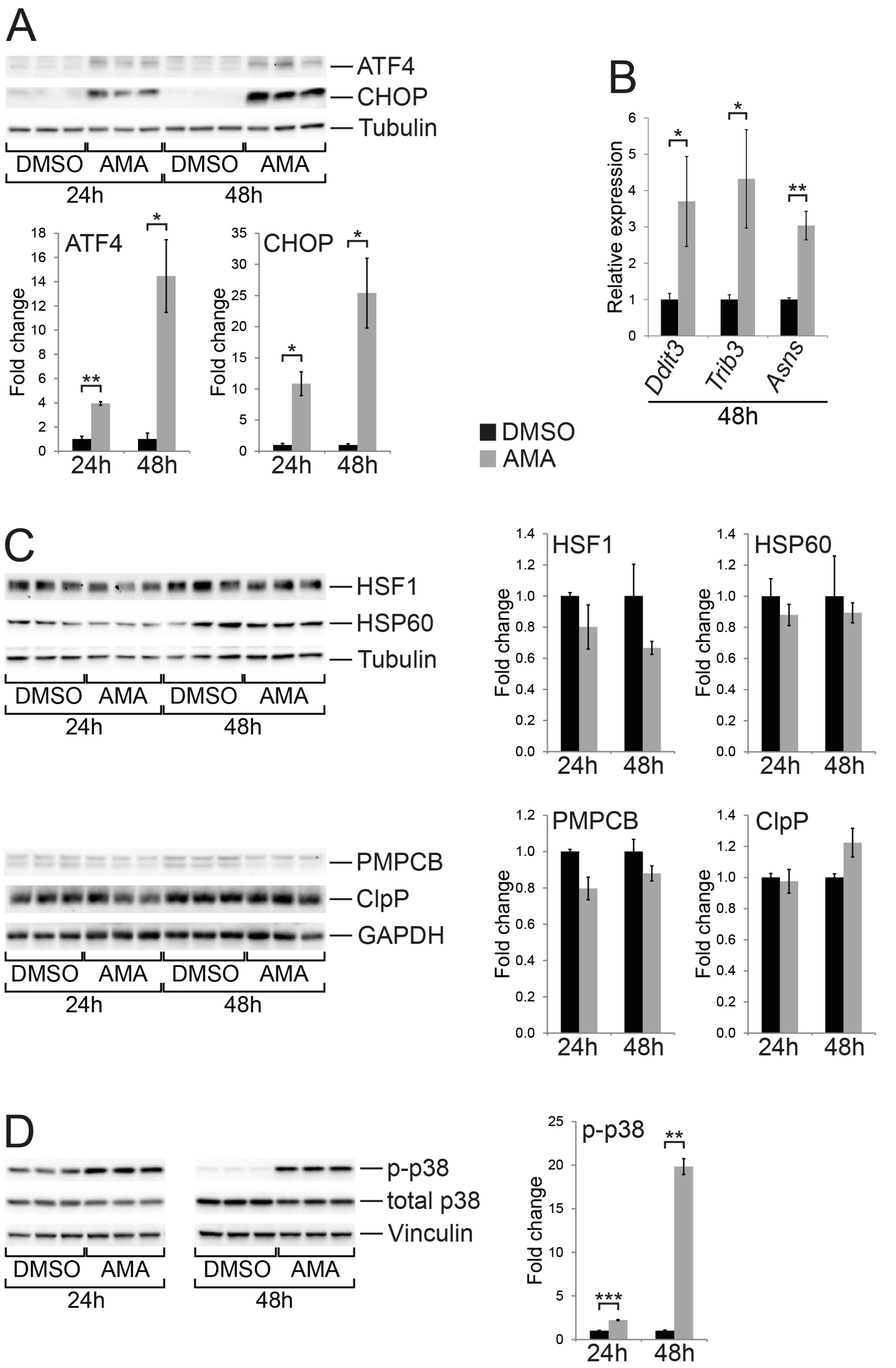

Supplementary Figure 10. Characterization of stress response pathways in HL-1 cells treated with DMSO or AMA (50 µM) for 24h or 48h. (A) Western blots showing protein expression of ATF4 and CHOP, core transcription factors of the ISR. (B) qRT-PCR data showing RNA expression of the ISR target genes *Ddit3* (encoding CHOP), *Trib3* and *Asns*. (C) Western blots showing protein expression of HSF1, a key transcription factor regulating the HSR, as well as HSP60, PMPCB and ClpP, proteins involved in the UPR^{mt}. (D) Western blots showing phosphorylation of the stress responsive p38 MAP kinase. (**P*<0.05, ***P*<0.01, ****P*<0.001, n=3 wells per treatment)

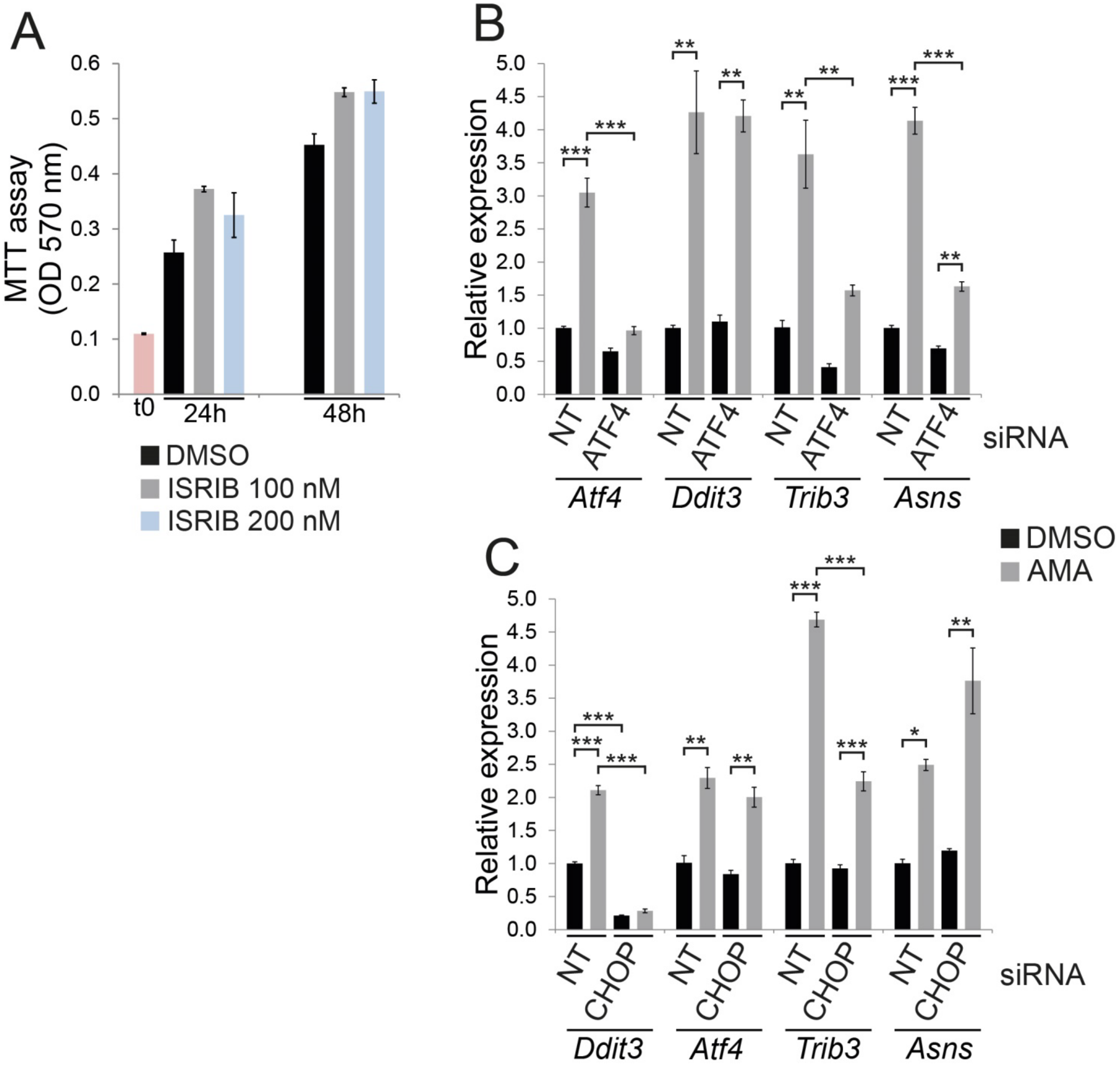

Supplementary Figure 11. Inhibition of the ISR in H9c2 cells treated with antimycin A. (A) MTT assays of H9c2 cells treated with DMSO and two different concentrations of ISRIB for 24h and 48h (t0 represents MTT values at the onset of treatment). (B) qRT-PCR analyses showing RNA expression of genes involved in the ISR in H9c2 cells transfected with non-target (NT) or ATF4 siRNA and subsequently treated with DMSO or AMA (50 µM) for 48h. *Atf4* was included to evaluate gene knockdown on RNA level. *Ddit3* encodes CHOP whereas *Trib3* and *Asns* are target genes known to be induced by the ISR. (C) qRT-PCR analyses showing RNA expression of genes involved in the ISR in H9c2 cells transfected with non-target (NT) or CHOP siRNA and subsequently treated with DMSO or AMA for 48h. *Ddit3* encodes CHOP and verifies gene knockdown on RNA level (other genes see (B)). (**P*<0.05, ***P*<0.01, ****P*<0.001, n=3 wells per treatment in (A) – (C))

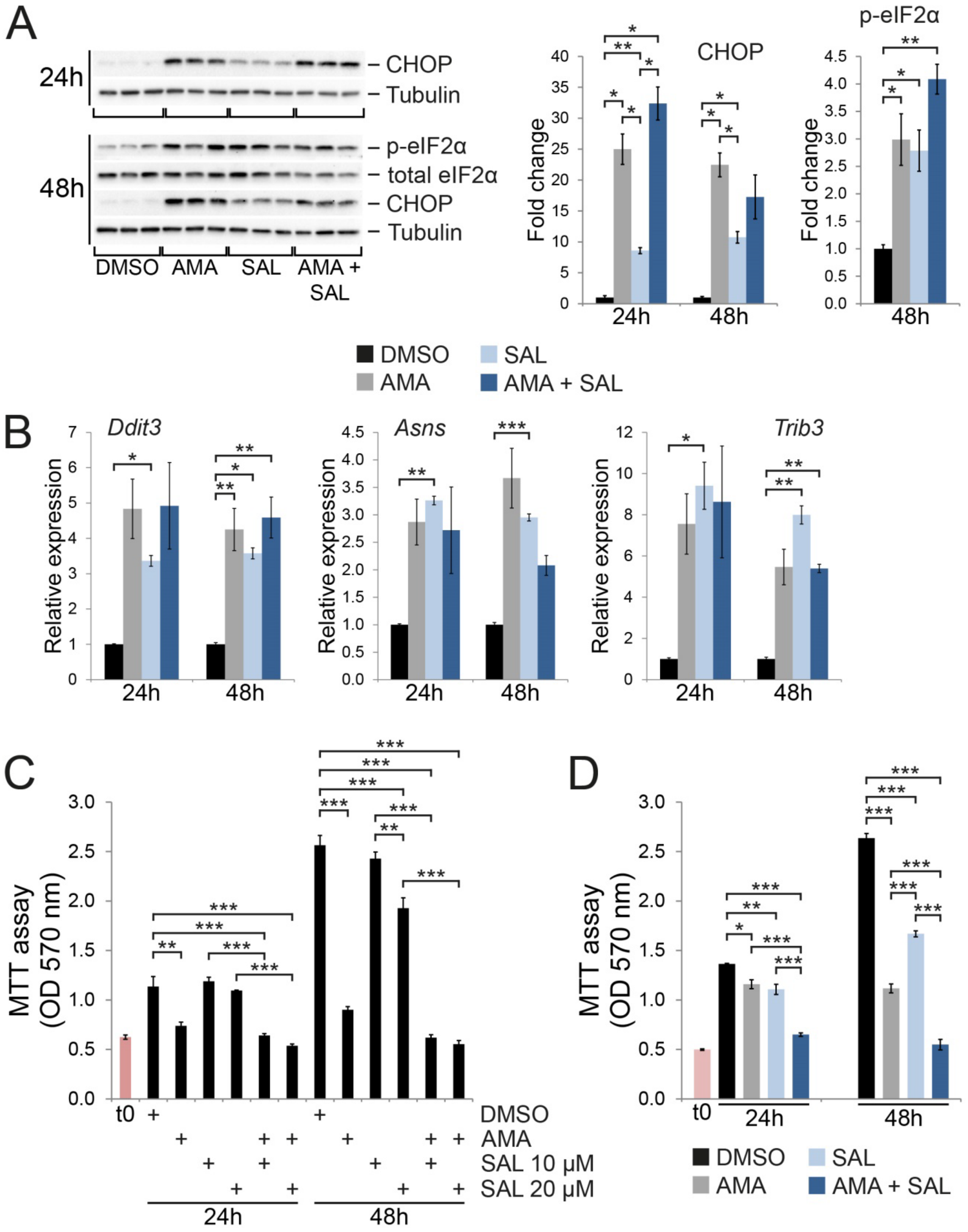

Supplementary Figure 12. Pharmacological activation of the ISR does not rescue growth of HL-1 cells upon antimycin A treatment. (A) Western blots showing phosphorylation of eIF2α and CHOP expression in HL-1 cells treated with DMSO, AMA (50 µM), Salubrinal (SAL, 20 µM) or a combination of AMA and SAL for 24h and 48h. Salubrinal activates the ISR evident as increased CHOP expression and eIF2α phosphorylation when compared to DMSO. Stress induced expression of CHOP by AMA exceeds the effect of Salubrinal, however. (B) qRT-PCR analyses of HL-1 cells

treated as outlined in (A) to detect RNA expression of *Ddit3* (encoding CHOP) as well as the known ISR target genes *Asns* and *Trib3*. Salubrinal induces ISR gene expression to a similar extent as AMA. (C) MTT assays of HL-1 cells treated with DMSO, AMA (50 µM), two different concentrations of Salubrinal and the respective combinations of AMA and SAL for 24h and 48h. Co-treatment with Salubrinal does not alter the growth inhibitory effect of AMA. High concentration of Salubrinal by itself induces a mild growth inhibition after 48h. (D) MTT assays of HL-1 cells treated with DMSO, AMA (50 µM), Salubrinal (20 µM) and a combinations of AMA and SAL for 24h and 48h. SAL and AMA + SAL treated cells have furthermore been preincubated with Salubrinal (20 µM) for 24h in order to activate the ISR prior to the addition of AMA. SAL preincubation followed by AMA + SAL treatment worsen cell growth compared to AMA alone and long term SAL treatment has a growth inhibitory effect by itself. (**P*<0.05, ***P*<0.01, ****P*<0.001, n=3 wells per treatment in (A) – (D))

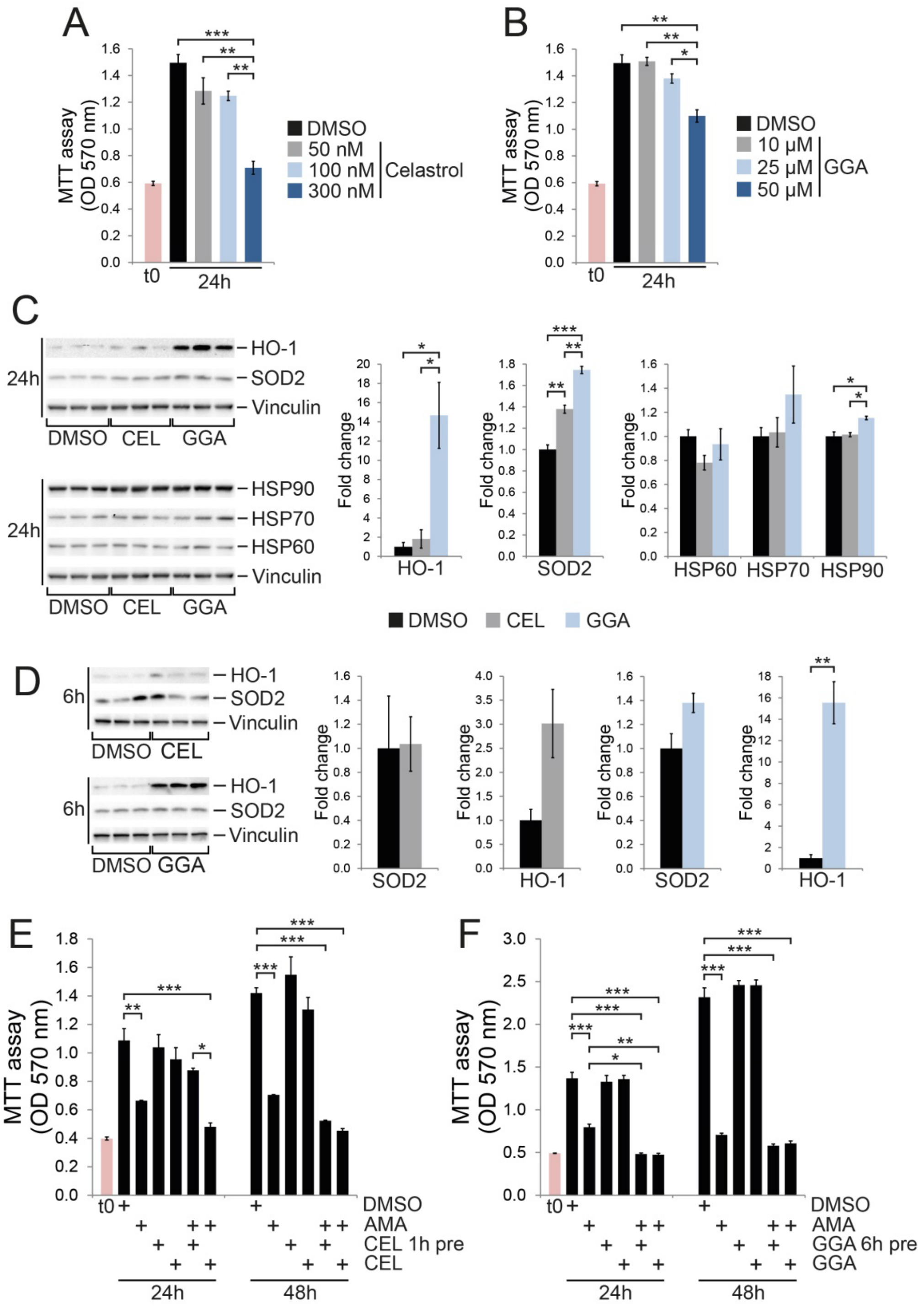

Supplementary Figure 13. Celastrol and Geranylgeranyl acetone do not rescue growth of HL-1 cells upon antimycin A treatment. (A) MTT assays of HL-1 cells treated with DMSO and three different concentrations of Celastrol or (B) Geranylgeranyl acetone (GGA) for 24h. (C) Western blots showing protein expression of HO-1 and SOD2 as well as various chaperones (HSP60, HSP70 and HSP90) in HL-1 cells treated with Celastrol (CEL, 100 nM) and GGA (25 μM) for 24h. (D) Western blots showing protein expression of HO-1 and SOD2 in HL-1 cells treated with Celastrol

(CEL, 100 nM) and GGA (25 μM) for 6h. (E) MTT assays of HL-1 cells treated with DMSO, AMA (50 μM), Celastrol (100 nM) or a combination of AMA and CEL for 24h and 48h. Some wells were pre-incubated with CEL for 1h (CEL 1h pre) in order to activate a stress response prior to AMA treatment, which was subsequently added without CEL. In other wells AMA and CEL were added simultaneously without CEL pre-incubation. Co-treatment with Celastrol does not alter the growth inhibitory effect of AMA in HL-1 cells after 48h whereas a mild protection is caused by CEL pre-incubation after 24h. (F) MTT assays of HL-1 cells treated with DMSO, AMA (50 μM), GGA (25 μM) or a combination of AMA and GGA for 24h and 48h. Some wells were pre-incubated with GGA for 6h (GGA 6h pre) in order to activate a stress response prior to AMA treatment, which was subsequently added without GGA. In other wells AMA and GGA were added simultaneously without GGA pre-incubation. Neither co- nor pretreatment with GGA rescue the growth inhibitory effect of AMA in HL-1 cells but instead further inhibit cell growth over the first 24h when compared to AMA alone. (*$P<0.05$, **$P<0.01$, ***$P<0.001$, n=3 wells per treatment in (A) – (F), t0 represents MTT values at the onset of treatment in (A), (B), (E) and (F))

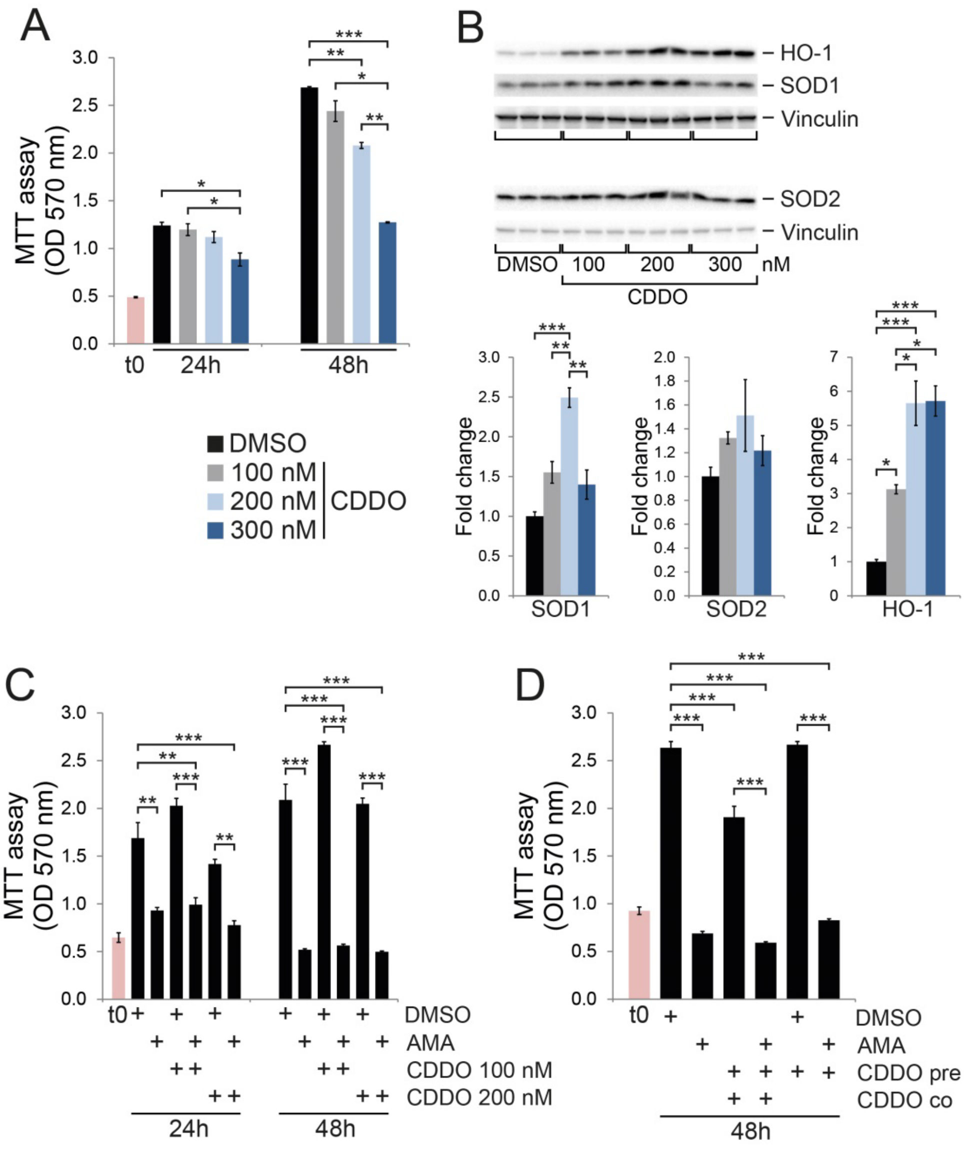

Supplementary Figure 14. CDDO methyl ester does not rescue growth of HL-1 cells upon antimycin A treatment. (A) MTT assays of HL-1 cells treated with DMSO or three different concentrations of CDDO for 24h and 48h. (B) Western blots showing protein expression of HO-1, SOD1 and SOD2 in HL-1 cells treated with three different concentrations of CDDO for 24h. (C) MTT assays of HL-1 cells treated with DMSO, AMA (50 µM), two different concentrations of CDDO or a combination of AMA and CDDO for 24h and 48h (AMA and CDDO were added simultaneously). Co-treatment with CDDO does not rescue the growth inhibitory effect of AMA in HL-1 cells. (D) MTT assays of HL-1 cells treated with DMSO, AMA (50 µM), CDDO (200 nM) or a combination of AMA and CDDO. Some wells were pre-incubated with CDDO for 24h (CDDO pre) followed by treatment with DMSO, AMA or AMA plus CDDO (CDDO co) for 48h. Neither co- nor pretreatment with CDDO rescue the growth inhibitory effect of AMA in HL-1 cells. (**P*<0.05, ***P*<0.01, ****P*<0.001, n=3 wells per treatment in (A) – (D), t0 represents MTT values at the onset of treatment in (A), (C) and (D))

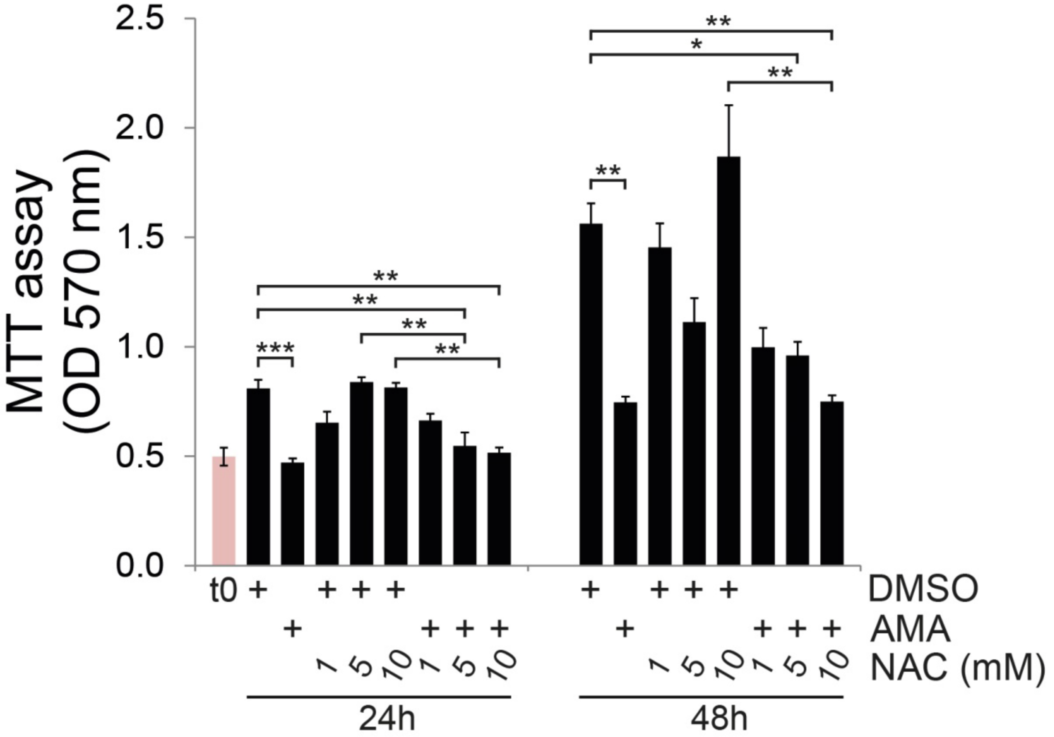

Supplementary Figure 15. N-acetylcysteine does not rescue growth of HL-1 cells upon antimycin A treatment. MTT assays of HL-1 cells treated with DMSO or AMA (50 μM) and co-incubated with three different concentrations of the ROS scavenger N-acetylcysteine (NAC) for 24h and 48h. Co-treatment with NAC does not rescue the growth inhibitory effect of AMA in HL-1 cells. (**P*<0.05, ***P*<0.01, ****P*<0.001, n=3 wells per treatment, t0 represents MTT values at the onset of treatment)

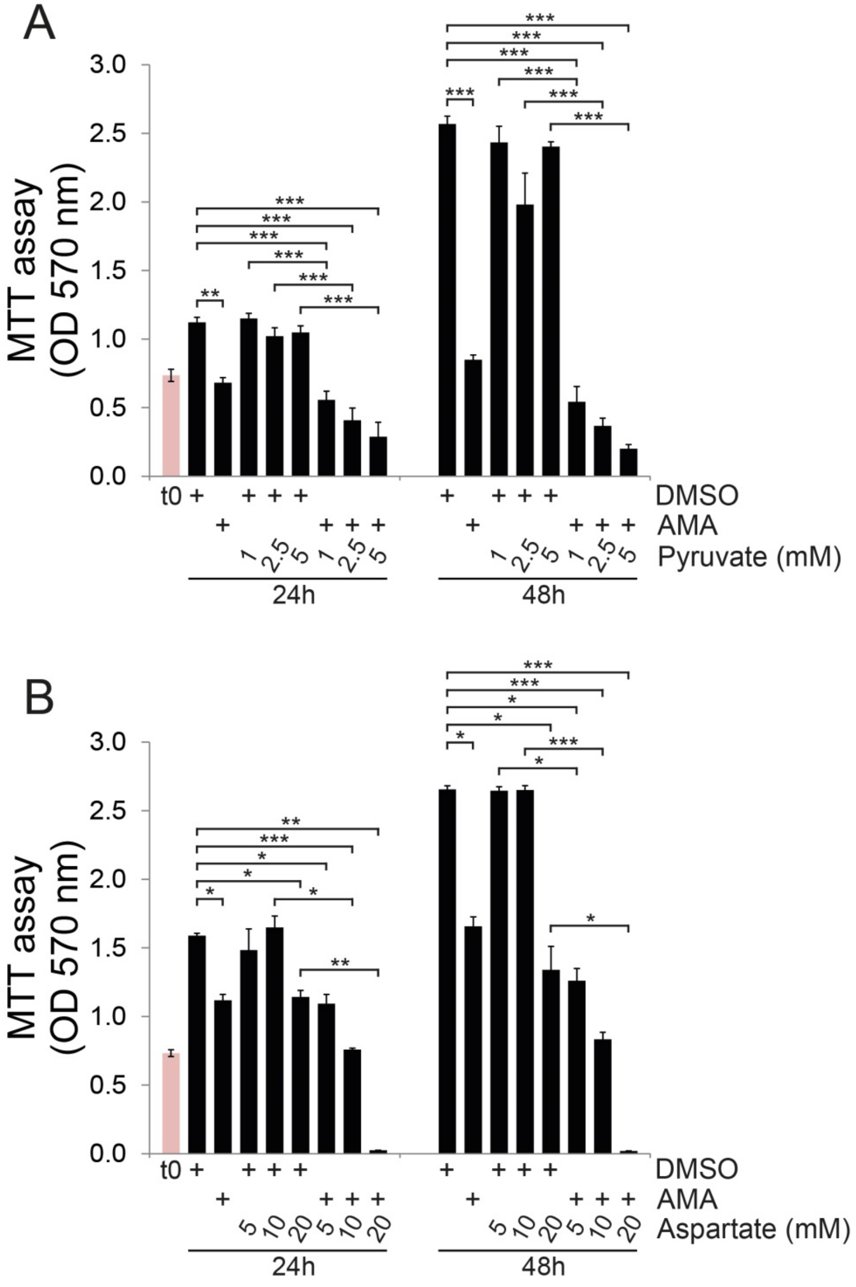

Supplementary Figure 16. Pyruvate and aspartate supplementation do not rescue growth of HL-1 cells upon antimycin A treatment. (A) MTT assays of HL-1 cells treated with DMSO or AMA (50 µM) and cultured in medium supplemented with three different concentrations of pyruvate for 24h and 48h. Co-treatment with pyruvate does not rescue the growth inhibitory effect of AMA in HL-1 cells. (B) MTT assays of HL-1 cells treated with DMSO or AMA (50 µM) and cultured in medium supplemented with three different concentrations of aspartate for 24h and 48h. Co-treatment with aspartate does not rescue the growth inhibitory effect of AMA in HL-1 cells. High concentration of aspartate by itself impairs cell growth and survival likely due to acidification of the culture medium. (*P<0.05, **P<0.01, ***P<0.001, n=3 wells per treatment in (A) and (B), t0 represents MTT values at the onset of treatment in (A) and (B))

Supplementary Tables

Supplementary Table 1: Primary antibodies used for Western blot analyses.

Antigen	Manufacturer	Order number	Dilution
ATF4	Cell Signaling	#11815	1:1000
CHOP	Cell Signaling	#5554	1:1000
cleaved caspase 3	Cell Signaling	#9661	1:1000
ClpP	Sigma Aldrich	WH0008192M1	1:1000
phospho-eIF2α	Cell Signaling	#3597	1:1000
total eIF2α	Cell Signaling	#2103	1:1000
GADD34	Santa Cruz	sc-8327	1:1000
GAPDH	Thermo Fisher	MA1-16757	1:50000
HO-1	Abcam	ab13243	1:2000
HSF1	Cell Signaling	#4356	1:1000
HSP60	Abcam	ab46798	1:20000
HSP70	Cell Signaling	#4872	1:1000
HSP90	Cell Signaling	#4877	1:1000
total JNK	Santa Cruz	sc-571	1:1000
NRF1	Abcam	ab175932	1:5000
NRF2	Santa Cruz	sc-722	1:500
phospho-p38	Cell Signaling	#4511	1:1000
total p38	Cell Signaling	#8690	1:1000
PMPCB	Proteintech	16064-1-AP	1:1000
PRDX3	Abcam	ab73349	1:2000
PRDX-SO3	Abcam	ab16830	1:2000
phospho-SAPK/JNK	Cell Signaling	#9251	1:1000
SOD1	Abcam	ab16831	1:2000
SOD2	Abcam	ab13533	1:5000
Tubulin	Sigma Aldrich	T9026	1:5000
Vinculin	Sigma Aldrich	V9131	1:5000

Supplementary Table 2: Sequences of primers used for qRT-PCR analyses.

Gene	Forward Primer	Reverse Primer
**Rat primers (for H9c2 cells)**		
Atf4	5′- CGGCCACCATGGCGTATTA -3′	5′- TTGTCCGTTACAGCAACGCT -3′
Ddit3	5′- CTATATCTCATCCCCAGGAAAC -3′	5′- CATAGAACTCTGACTGGAATC -3′
Asns	5′- GCTGTTTTGGCTTCCACCGGC -3′	5′- TGGTTTTCTCGATGCCGCCTTTG -3′
Trib3	5′- GTCGATTTGTCTTCAGCAAC -3′	5′- CTGAGTATCTCTGGTCCCAC -3′
Nfe2l2	5′- CACATCCAGACAGACACCAGT -3′	5′- CTACAAATGGGAATGTCTCTGC -3′
Polr2a	5′- GAGAAGCTGGTCCTTCGTATC -3′	5′- GCATGTTGGACTCAATGCACC -3′
**Mouse primers (for HL-1 cells)**		
Ddit3	5′- CTATATCTCATCCCCAGGAAAC -3′	5′- CATAGAACTCTGACTGGAATC -3′
Asns	5′- TCCAAGTATATTCGGAAGAAC -3′	5′- TCCAAGTATATTCGGAAGAAC -3′
Trib3	5′- GTCGCTTTGTCTTCAGCAAC -3′	5′- GTCGCTTTGTCTTCAGCAAC -3′
Polr2a	5′- GAGAAGCTGGTCCTTCGAATC -3′	5′- GCATGTTGGACTCAATGCATC -3′
